# Systematic Analysis of c-di-GMP Signaling Mechanisms and Biological Functions in Dickeya zeae EC1

**DOI:** 10.1128/mBio.02993-20

**Published:** 2020-12-01

**Authors:** Yufan Chen, Jianuan Zhou, Mingfa Lv, Zhibin Liang, Matthew R. Parsek, Lian-hui Zhang

**Affiliations:** a Guangdong Province Key Laboratory of Microbial Signals and Disease Control, South China Agricultural University, Guangzhou, People’s Republic of China; b Integrative Microbiology Research Centre, South China Agricultural University, Guangzhou, People’s Republic of China; c Department of Microbiology, University of Washington, Seattle, Washington, USA; University of Minnesota Medical School

**Keywords:** *Dickeya zeae*, c-di-GMP, biofilm formation, swimming motility, consecutive in-frame deletion, sessile-to-motile transition, biofilms, plant pathogens, quorum sensing, rhizosphere-inhabiting microbes

## Abstract

Dickeya zeae is an important and aggressive bacterial phytopathogen that can cause substantial economic losses in banana and rice plantations. We previously showed that c-di-GMP signaling proteins (cyclases/phosphodiesterases) in D. zeae strain EC1 play a significant role in the bacterial sessile-to-motile transition. To determine whether there is any synergistic effect among these c-di-GMP signaling proteins, we prepared a series of mutant strains by generating consecutive in-frame deletions of the genes encoding diguanylate cyclases (which make c-di-GMP) and phosphodiesterases (which break down c-di-GMP), respectively, using EC1 as a parental strain. The results showed that the complete deletion of all the putative diguanylate cyclases resulted in significantly increased bacterial motility and abrogated biofilm formation but did not appear to affect pathogenicity and virulence factor production. In contrast, the deletion of all the c-di-GMP phosphodiesterase genes disabled motility and prevented the invasion of EC1 into rice seeds. By measuring the c-di-GMP concentrations and swimming motility of all the mutants, we propose that c-di-GMP controlled swimming behavior through a multitiered program in a c-di-GMP concentration-dependent manner, which could be described as an L-shaped regression curve. These features are quite different from those that have been shown for other bacterial species such as *Salmonella* and Caulobacter crescentus. Further analysis identified three c-di-GMP signaling proteins, i.e., PDE10355, DGC14945, and PDE14950, that play dominant roles in influencing the global c-di-GMP pool of strain EC1. The findings from this study highlight the complexity and plasticity of c-di-GMP regulatory circuits in different bacterial species.

## Abstract (precis)

Dickeya zeae is the etiological agent of bacterial foot rot disease, which can cause massive economic losses in banana and rice plantations. Genome sequence analysis showed that *D. zeae* strain EC1 contains multiple c-di-GMP turnover genes, but their roles and regulatory mechanisms in bacterial physiology and virulence remain vague. By generating consecutive in-frame deletion mutants of the genes encoding c-di-GMP biosynthesis and degradation, respectively, we analyzed the individual and collective impacts of these c-di-GMP metabolic genes on the c-di-GMP global pool, bacterial physiology, and virulence. The significance of our study is in identifying the mechanism of c-di-GMP signaling in strain EC1 more clearly, which expands the c-di-GMP regulating patterns in Gram-negative species. The methods and experimental designs in this research will provide a valuable reference for the exploration of the complex c-di-GMP regulation mechanisms in other bacteria.

## INTRODUCTION

*Dickeya* spp. are prominent plant-pathogenic bacterial species, which are problematic in the agricultural industry internationally (1). Among the *Dickeya* family members, Dickeya zeae is the causal agent of bacterial foot rot disease, causing significant economic losses in banana- and rice-producing agricultural regions in recent years. The pathogen was known previously as Erwinia chrysanthemi pv*. zeae* but was reclassified as a member of the new genus *Dickeya* in 2005 (2). The genomes of several species and strains of *Dickeya* have been sequenced and deposited in GenBank (3), facilitating the identification and characterization of the genes and molecular mechanisms associated with bacterial physiology and virulence.

Much of what is known about the virulence and pathogenesis of the *Dickeya* genus was largely obtained through the characterization of Dickeya dadantii over the last 30 years (4). D. dadantii is known to produce a range of virulence factors, including plant cell wall-degrading exoenzymes (5–7); an iron assimilation system; the blue pigment indigoidine, which serves as an antioxidant (8, 9); as well as a type III secretion system (10, 11). Genome analysis and biochemical characterization showed that most, if not all, of these virulence traits are conserved in D. zeae (12). In addition, a family of phytotoxins and antibiotics known as zeamines was characterized as a key feature of *D. zeae* strain EC1, which is the causal agent of rice foot rot disease (13). Comparative genomic analysis revealed that the zeamine biosynthetic gene cluster is present only in *D. zeae* strains isolated from rice and in some strains of the related pathogen Dickeya solani (12).

In addition, several regulatory mechanisms associated with *D. zeae* virulence have been uncovered in recent years, including the transcription factors SlyA and Fis (14, 15); a two-component regulatory system, VfmHI (16); an acyl-homoserine lactone (AHL)-mediated quorum sensing (QS) system (17); a polyamine-mediated host-pathogen communication system (18); and the bacterial second messenger cyclic di-GMP (c-di-GMP) (19). These findings present both a framework and useful clues for dissecting the molecular mechanisms and signaling networks by which *D. zeae* modulates its physiology and virulence.

c-di-GMP has emerged as a universal second messenger that is widely distributed within the bacterial kingdom (20–22). c-di-GMP has been shown to regulate cell motility, biofilm formation, the production of virulence factors, cell differentiation, the cell cycle, and other cellular processes in a wide range of species (22–24). Cellular c-di-GMP levels are modulated by diguanylate cyclases (DGCs), which are characterized by GGDEF domains and are involved in its synthesis, and phosphodiesterases (PDEs), which harbor either EAL or HD-GYP catalytic domains that catalyze c-di-GMP degradation (25–27). Typically, most bacterial species contain multiple DGCs and PDEs as well as different types of c-di-GMP receptors, indicating the importance of c-di-GMP for bacterial physiology (22).

However, much of the current knowledge regarding the physiological roles of c-di-GMP came from investigations of individual c-di-GMP signaling genes. Given that multiple, potential c-di-GMP signaling proteins are generally encoded by a single bacterial genome, either mutation or overexpression of individual c-di-GMP signaling genes sometimes limits our ability to discern their role due to potential compensatory or redundant activity from the remaining c-di-GMP signaling proteins. One approach that researchers have taken to investigate c-di-GMP signaling in species containing multiple DGC and PDE genes is to generate strains bearing multiple mutations in DGC-encoding genes that cannot make c-di-GMP (28–30). Using this strategy, Abel et al. found that for Caulobacter crescentus, a strain unable to make c-di-GMP exhibited an absence of flagellar motility and was unable to attach to surfaces (29). However, c-di-GMP DGC-null strains of *Salmonella* and Sinorhizobium meliloti exhibited motility and biofilm behaviors similar to those of the wild type (WT) (30, 31). These findings highlight the variable role that c-di-GMP signaling may play in the biology of different bacterial species.

*D. zeae* strain EC1 encodes 19 putative c-di-GMP signaling proteins, including 12 GGDEF-containing proteins, 4 EAL/HD-GYP domain proteins, as well as 3 proteins that have both domains. In our previous study, individual mutations in all GGDEF/EAL/HD-GYP domain-containing genes were constructed, and virulence-associated phenotypes were assessed (19). We showed that most of the individual mutant strains were altered in biofilm formation and flagellum-mediated motility without affecting toxin zeamine production and virulence (19). However, our previous analysis of individual mutants was limited given the presence of multiple, chromosomally encoded c-di-GMP signaling proteins. To address this, we systematically constructed a c-di-GMP-null mutant strain (which we termed cdG^0^) by deleting all the genes encoding GGDEF domain-containing proteins in *D. zeae* EC1. The results showed that the cdG^0^ mutant strain (15ΔDGC) exhibited increased flagellum-mediated motility and was unable to form a visible biofilm but remained fully virulent to rice seeds. In addition, to gain insight into high-c-di-GMP physiology, all the PDE-related genes were also deleted consecutively. The results showed that a completely PDE-null mutant strain (containing 7 PDE deletions) generally displayed the opposite phenotypes of the cdG^0^ mutant. The PDE-null mutant background displayed a complete defect in swimming motility and showed attenuated virulence but exhibited hyperbiofilm formation. In addition, we observed that c-di-GMP levels tuned the swimming motility response. Finally, our analysis allowed us to identify the dominant “housekeeping” DGC and PDE genes that affect the global c-di-GMP level in *D. zeae* under laboratory conditions.

## RESULTS

### Bioinformatic analysis and generation of deletion mutant strains.

**FIG 1:**
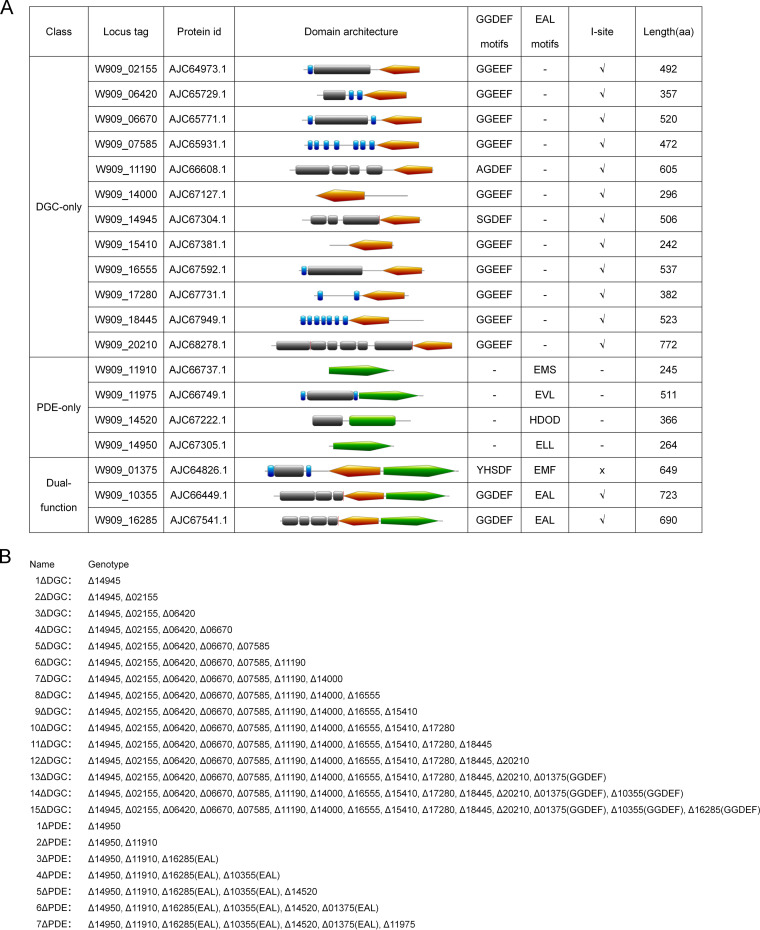
Domain structures of c-di-GMP metabolic enzymes and related mutants generated in this study. (A) Predicted domain architectures and consensus sequence motifs. Orange, GGDEF domain; green, EAL/HD-GYP domain; blue bars, predicted transmembrane domain; gray boxes, other domains. (For more details, see reference 19.) Three proteins contain nonclassical GG(D/E)EF domains, i.e., W909_11190, W909_14945, and W909_01375. Two EAL domain-containing proteins (W909_11910 and W909_01375) lack the conserved key motif (EXLXR). aa, amino acids. (B) Mutants and genotypes used in this study.

Our previous study showed that individual mutations for several genes encoding DGC enzymes in *D. zeae* strain EC1 resulted in decreased biofilm formation and increased motility compared to the wild type, while the deletion of some individual PDE genes led to the expected opposite phenotypes (19). Bioinformatics analysis revealed that *D. zeae* strain EC1 potentially encodes 12 GGDEF domain proteins, 4 EAL/HD-GYP proteins, and 3 dual-functional-domain proteins (Fig. 1A). To assess the role of the c-di-GMP global pool in *D. zeae* physiology and virulence, these DGC or PDE genes were systematically deleted in frame to generate DGC- and PDE-null mutant backgrounds (Fig. 1B). For three genes (*W909_01375*, *W909_10355*, and *W909_16285*) that encode dual-function proteins (Fig. 1A), only the GGDEF or EAL domains of these genes were deleted in frame (Fig. 1B).

### Characterization of the cdG^0^ strain reveals changes in bacterial motility and biofilm formation.

**FIG 2:**
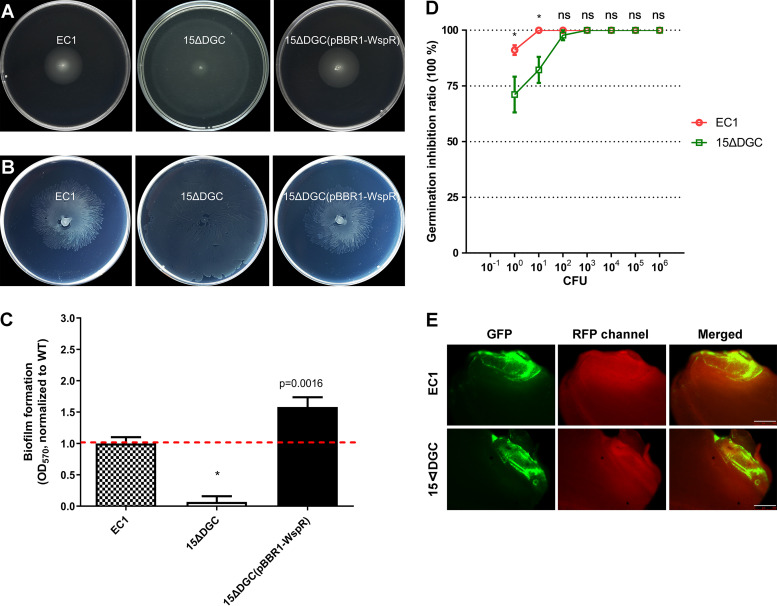
Phenotypes of c-di-GMP-free mutant 15ΔDGC. (A) Swimming motility. (B) Swarming motility. (C) Biofilm formation in SOB plus 1% sucrose (SOBS) medium. *, *P* < 0.001 (by Student’s *t* test) (*n* ≥ 3 independent experiments). (D) Virulence assay on rice seed germination. Seven dilution gradients of bacterial cultures were cocultured with 15 rice seeds at room temperature for 6 h before being washed with sterilized water, and rice seeds were then germinated at 28°C under 16 h of light and 8 h of dark for 1 week. *, *P* < 0.001; ns, not significant (*P* > 0.05) (by two-way analysis of variance [ANOVA] with multiple comparisons) (*n* ≥ 3 independent experiments). (E) Fluorescence inverted microscopy observation of colonization. Rice seeds were incubated with strain EC1 and mutant 15ΔDGC carrying a plasmid expressing GFP for 6 h and transferred to moistened filter papers at 28°C for 40 h before fluorescence microscope observation. Bars = 500 μm.

Previous studies showed that c-di-GMP-free (cdG^0^) mutants were either impaired or comparable for cell motility of C. crescentus and *Salmonella* (29, 31), which appears contradictory to the general concept that a low c-di-GMP concentration increases cell motility (21, 22). To determine if c-di-GMP had similar effects on *D. zeae* physiology, we initially analyzed our cdG^0^ background. Different from the *Salmonella* and C. crescentus findings, we found drastic increases in both swimming and swarming motility in the cdG^0^ strain (Fig. 2A and B; see also Fig. S1A and B in the supplemental material). This strain also demonstrated a much-reduced capacity to form biofilms (Fig. 2C) but did not show a difference in the production of exoenzymes and zeamines (Fig. S2). Complementation of the mutant with a heterologously expressed DGC gene (*wspR*) from Pseudomonas aeruginosa restored both biofilm and motility to levels comparable to those of the wild type (32) (Fig. 2 and Fig. S1A and B). The rice seed germination assay showed that the cdG^0^ mutant strain showed levels of rice seed germination inhibition comparable to those of the wild-type strain EC1 except at the low end of the inoculum (Fig. 2D).

10.1128/mBio.02993-20.1 FIG S1 Motility analysis and growth curves of EC1 and its derivatives. (A and B) Statistical analysis of swimming and swarming motility of EC1 and derivatives described in the legends of Fig. 2 and 4. For easy comparison, the motility of each mutant was normalized to that of wild-type EC1, which was set to a value of 1. Each sample was statistically compared to every other sample; the same lowercase letters indicate samples that are not significantly different, and different letters indicate significant differences (*P* < 0.0001), as determined by one-way ANOVA with a multiple-comparison test. (C and D) Growth curves of EC1 and its derivatives. The growth curves of all DGC mutant intermediates (C) and all PDE intermediates (D) in LB medium were tested at 28°C in a low-intensity shaking model using the Bioscreen-C automated growth curve analysis system (OY Growth Curves AB, Ltd., Finland). The optical density at 600 nm of the bacterial culture was measured every 4 h until 36 h. The experiments were repeated twice with 3 replicates. Download FIG S1, TIF file, 2.1 MB. Copyright © 2020 Chen et al. 2020 Chen et al. This content is distributed under the terms of the Creative Commons Attribution 4.0 International license.

10.1128/mBio.02993-20.2 FIG S2 Production of exoenzymes and zeamines is not regulated by the c-di-GMP global pool in EC1. (A to D) The production of four types of exoenzymes (Pel [A], Peh [B], Prt [C], and Cel [D]) in mutants lacking either all c-di-GMP synthetases or hydrolases and their heterocomplementary strains was assayed. Strains were cultured in LB medium until the OD_600_ reached 1.5, and 20 μl of the bacterial culture was added to the wells and incubated at 28°C for at least 11 h (see Materials and Methods). 1, EC1 (wild type); 2, 15ΔDGC; 3, 7ΔPDE; 4, EC1(pBBR1MCS4); 5, 15ΔDGC(pBBR1MCS4); 6, 7ΔPDE(pBBR1MCS4); 7, 15ΔDGC(pBBR1-WspR^stalk-GGDEF^); 8, 7ΔPDE(pBBR1-RocR); 9, EC1 (wild type); 10, LB medium (negative control). (E) The production of zeamines of mutants lacking either all c-di-GMP synthetases or hydrolases and their heterocomplementary strains was assayed. The activity of antibiotics produced by mutants with zero and the maximum concentration of c-di-GMP and their heterocomplementary strains is indicated relative to that of wild-type EC1. The dotted line indicates the wild-type antibiotic level. For easy comparison, the final results for each mutant were normalized to that of wild-type EC1, which was set to a value of 1. An asterisk indicates statistical significance by Student’s *t* test (*P* < 0.01), and “ns” indicates a *P* value of >0.05, in at least 3 technical replicates. Error bars indicate standard deviations. Download FIG S2, JPG file, 2.7 MB. Copyright © 2020 Chen et al. 2020 Chen et al. This content is distributed under the terms of the Creative Commons Attribution 4.0 International license.

To examine *D. zeae* invasion of rice seeds (an important aspect of pathogenesis), we introduced a plasmid expressing the *gfp* gene as a visible marker into the wild-type and the cdG^0^ mutant strains. Fluorescence microscopy was performed 40 h after inoculation, and the green fluorescent protein (GFP) fluorescence intensity in the regions of interest (ROIs) was then measured. We found that the mutant strain retained the ability to invade rice seeds (Fig. 2E and Fig. S3A).

10.1128/mBio.02993-20.3 FIG S3 Phenotypes of EC1 and its derivatives. (A) GFP intensity of regions of interest (ROI) for EC1 and its derivatives. For easy comparison, the GFP intensity of each mutant was normalized to that of wild-type EC1, which was set to a value of 1. Each sample was statistically compared to every other sample; the same lowercase letters indicate samples that are not significantly different, and different letters indicate significant differences (*P* < 0.0001), as determined by one-way ANOVA with a multiple-comparison test. (B) Δ*bcsA* represents the biofilm-negative strain. (C) A Δ*fliG* mutant served as a nonmotile control, which lost the colonization and evasion activities. (D) Seed germination assay results for EC1 and the Δ*bcsA* and Δ*fliG* mutants. * above the points for wild-type EC1 represents statistical analysis between EC1 and the Δ*bcsA* mutant, while ** above the points for the Δ*fliG* strain represents statistical analysis between EC1 and the Δ*fliG* mutant. *, *P* < 0.05; **, *P* < 0.001; ns, *P* > 0.05 (by two-way ANOVA with multiple comparisons) (*n* ≥ 2 independent experiments). (E) Rice seeds were infiltrated with the EC1, Δ*bcsA*, Δ*fliG*, and Δ*zmsA* strains carrying a GFP plasmid for 6 h and transferred to moistened filter papers at 28°C for 40 h before fluorescence microscopy observation. Download FIG S3, JPG file, 2.0 MB. Copyright © 2020 Chen et al. 2020 Chen et al. This content is distributed under the terms of the Creative Commons Attribution 4.0 International license.

**FIG 3:**
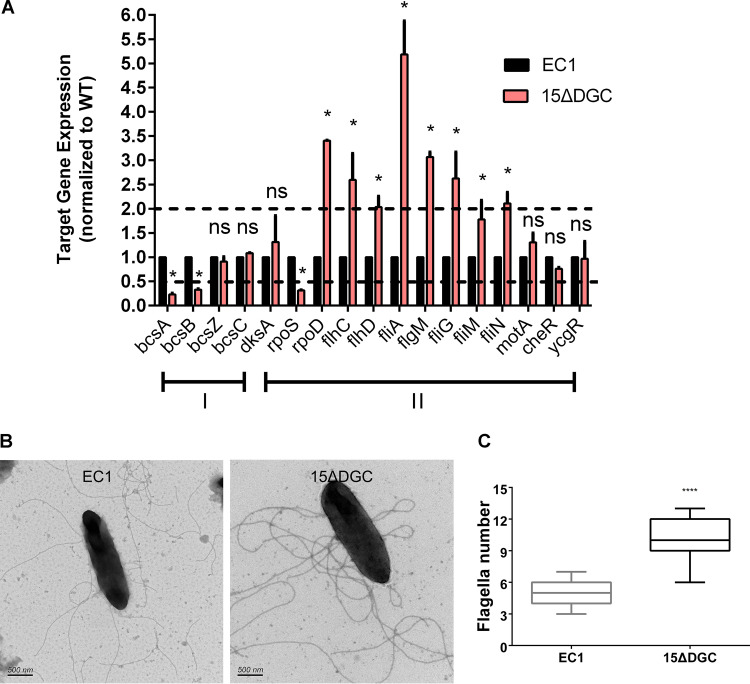
Quantitative reverse transcription-PCR (qRT-PCR) and transmission electron microscopy (TEM) analyses of wild-type EC1 and mutant 15ΔDGC. (A) Quantification of expression of the genes related to biofilm (I) and flagellar transcription (II) hierarchies by qRT-PCR. The following genes were quantified in this experiment: *bcsABZC*, exopolysaccharide cellulose genes (34); *dksA* and *rpoS*, encoding FlhDC transcriptional inhibition regulators (50, 62); *rpoD*, encoding a general sigma factor (σ^70^) that works in cooperation with FlhDC to direct RNA polymerase to transcribe class II genes (63); *flhC* and *flhD*, encoding a class I master regulator of the flagellar transcription hierarchy and a flagellar transcriptional activator (36); *fliA*, encoding a class II RNA polymerase sigma factor for a flagellar operon, which directly transcribes class III genes encoding flagellin, chemotactic signaling (Che), and motor (Mot) proteins (64); *flgM*, encoding the *fliA*-specific anti-sigma factor (65); *motA*, encoding a flagellar energy transduction protein (36); *cheR*, encoding a chemotaxis protein methyltransferase (66); and *ycgR*, encoding a flagellar brake protein (40). The dotted lines represent 2-fold and 50% gene expression levels compared to the wild type, which was set to a value of 1. *, *P* < 0.05; ns, *P* > 0.05 (by two-way ANOVA with multiple comparisons) (*n* ≥ 3 independent experiments). (B) Representative TEM of wild-type and 15ΔDGC cells after negative staining with 2% (wt/vol) phosphotungstic acid for 2 min. (C) Numbers of flagella of EC1 and 15ΔDGC. ****, *P* < 0.0001 (by Student’s unpaired *t* test).

Next, we quantitatively measured the transcriptional expression levels of a few known genes associated with biofilm formation and bacterial motility in the wild-type and mutant strains. Quantitative reverse transcription-PCR (qRT-PCR) results showed that the null mutant strain showed significantly decreased transcript levels of *bcsA* and *bcsB* (Fig. 3A), which encode key cellulose synthase enzymes involved in biofilm formation (33, 34). On the other hand, the transcript level of *rpoS*, a known repressor of the flagellar transcriptional hierarchy in *D. zeae* (35), was reduced by about 40%. Consistent with the above-described results, most of the class I (*rpoD*, *flhC*, and *flhD*), class II (*fliA*), and class III (*fliG* and *fliN*) flagellar gene transcripts (36) were elevated by at least 2-fold in the mutant strain compared to the wild type (Fig. 3A). Furthermore, transmission electron microscopy (TEM) analysis revealed that the *D. zeae* strain has a peritrichous flagellar arrangement, and the mutant strain 15ΔDGC generated more flagella than the wild type (Fig. 3B and C). These results suggest that the abolished biofilm formation and enhanced cell motility in the cdG^0^ mutant strain are due to changed expression patterns of the genes associated with bacterial motility and biofilm formation.

### A PDE-null strain exhibits completely impaired motility and a hyperbiofilm-forming phenotype.

**FIG 4:**
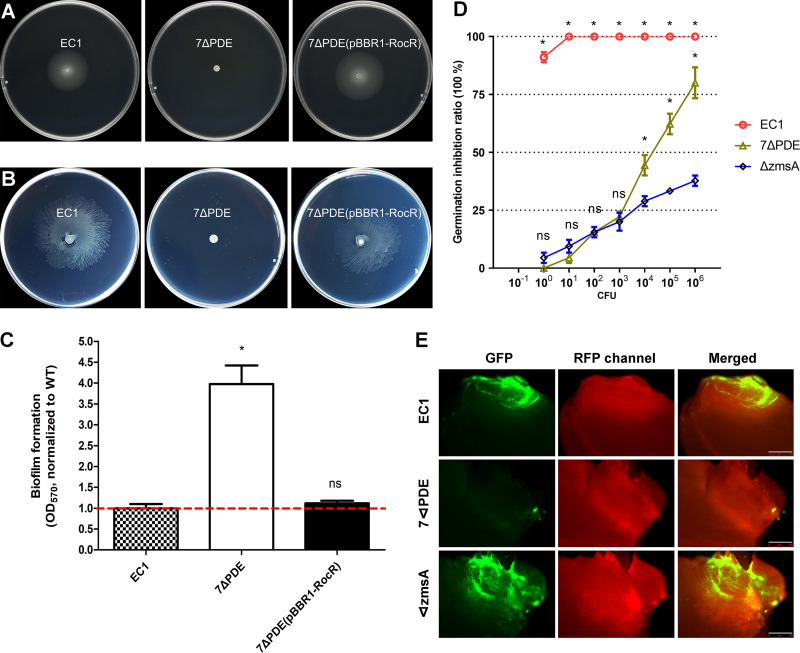
Phenotype assay of mutant 7ΔPDE. (A) Swimming motility. (B) Swarming motility. (C) Biofilm formation in SOBS medium. *, *P* < 0.001 (by Student’s *t* test) (*n* ≥ 3 independent experiments). (D) Virulence assay on rice seed germination. Asterisks above the points for wild-type EC1 represent statistical analyses of both EC1 versus 7ΔPDE and EC1 versus the Δ*zmsA* strain, while asterisks above the points for the 7ΔPDE strain represent statistical analysis between 7ΔPDE and the Δ*zmsA* strain. *, *P* < 0.0001; ns, *P* > 0.01 (by two-way ANOVA with multiple comparisons) (*n* ≥ 3 independent experiments). (E) Fluorescence inverted microscopy observation of colonization. Bars = 500 μm.

The above-described results showed that the cdG^0^ strain had stark phenotypic differences compared to similar c-di-GMP-null strains constructed for *Salmonella* and C. crescentus. These observations led us to examine a PDE-null strain (7ΔPDE). The PDE-null strain, similar to the cdG^0^ null mutant, displayed no significant changes in the production of exoenzymes and zeamines compared to the wild type (Fig. S2). However, the PDE-null strain was nonmotile and had a hyperbiofilm-forming phenotype (Fig. 4A to C and Fig. S1A and B), a direct contrast to the cdG^0^ strain. These phenotypes could be restored through the heterologous expression of the PDE enzyme RocR from P. aeruginosa PAO1 in the PDE-null background (37).

We next characterized virulence for the PDE-null strain using our rice seedling model system. Rice seeds were treated with various dilutions of the wild-type strain and the mutant strain for 6 h before being placed on damp filter papers for a germination assay in a growth chamber. These results showed that the PDE-null mutant strain was significantly attenuated in inhibiting rice seed germination compared to the wild type. This was comparable to a zeamine-free mutant, Δ*zmsA*, a negative control for the rice seed germination assay, at lower inocula (10^0^ to 10^3^) (13) (Fig. 4D). Consistent with its attenuated virulence, the PDE-null mutant cells were infrequently visible inside the rice grains (Fig. 4E and Fig. S3A). This was in contrast to the Δ*zmsA* strain, which showed marked GFP fluorescence inside the rice grains (Fig. 4E and Fig. S3A).

**FIG 5:**
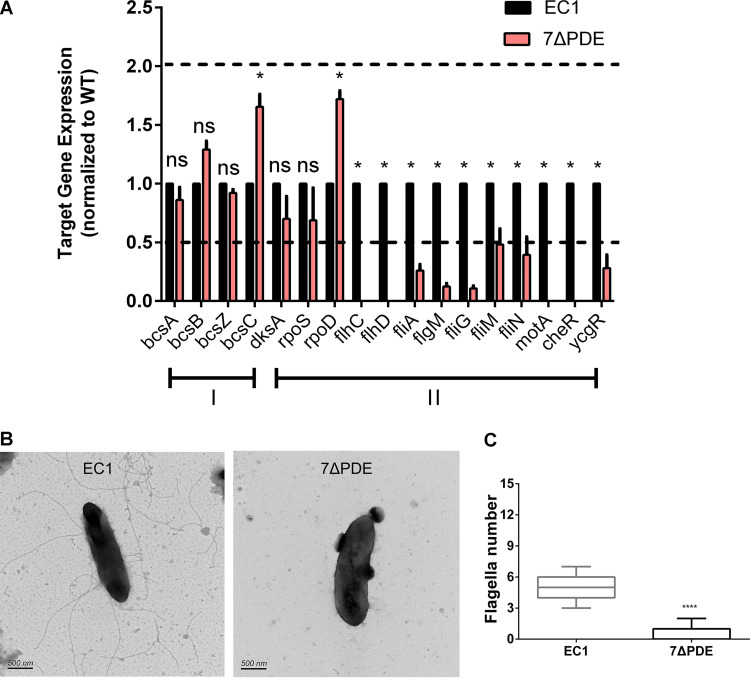
Quantitative RT-PCR and transmission electron microscopy analyses of wild-type EC1 and mutant 7ΔPDE. (A) Expression levels of the genes related to biofilm (I) and flagellar transcription (II) hierarchies by qRT-PCR. The dotted lines represent 2-fold and 50% gene expression levels compared to the wild-type, which was set to a value of 1. *, *P* < 0.01; ns, *P* > 0.01 (by two-way ANOVA with Bonferroni’s multiple-comparison test). (*n* ≥ 3 independent experiments). (B) Representative transmission electron microscopy graphs of wild-type EC1 and mutant 7ΔPDE cells after negative staining. (C) Numbers of flagella of EC1 and 7ΔPDE. ****, *P* < 0.0001 (by Student’s unpaired *t* test).

We then analyzed the transcriptional profiles for motility- and biofilm-related genes in the PDE-null strain. Transcript levels of the *bcs* operon were elevated slightly, whereas all the flagellar gene transcripts were decreased over 50%, in particular *flhC*, *flhD*, *motA*, and *cheR*, whose transcripts could not be detected by qRT-PCR (Fig. 5A). In accordance with the qRT-PCR results, TEM pictures of the negatively stained PDE-null mutant strain showed a significant defect in flagellar assembly (Fig. 5B and C).

Our data indicate that c-di-GMP-controlled flagellum-mediated motility and biofilm formation impact *D. zeae* virulence. Previous studies showed that *fliG* and *bcsA* are involved in bacterial motility and biofilm formation, respectively (38, 39). To directly examine which process impacts bacterial invasion and colonization, these two genes were deleted, and their phenotypes were evaluated (Fig. S3). A microscopic virulence assay was performed using the wild-type strain and two mutant strains, an isogenic nonmotile Δ*fliG* mutant and an isogenic biofilm-negative Δ*bcsA* mutant. After 40 h, scarcely any cells of the Δ*fliG* mutant were found inside the rice seeds, while the Δ*bcsA* mutant possessed the ability to invade the rice seeds (Fig. S3A, D, and E). Taken together, the above-described findings suggest that the second messenger c-di-GMP modulates virulence mainly through regulation of flagellum-mediated motility.

### c-di-GMP levels regulate swimming motility following an L-shaped regression curve.

**FIG 6:**
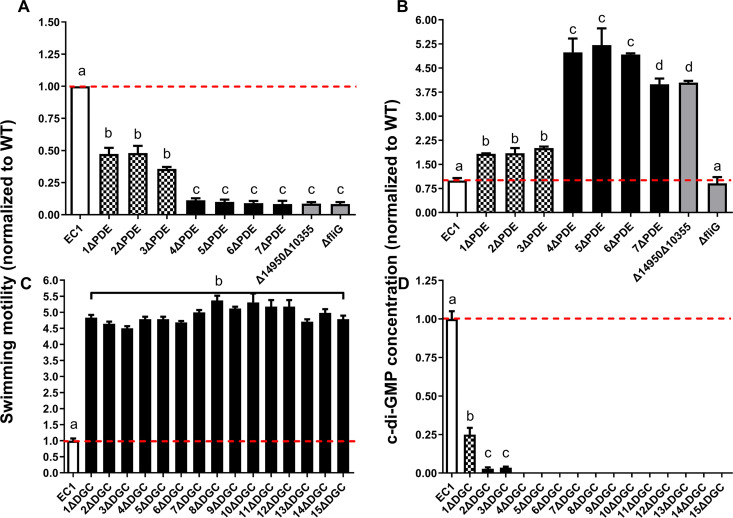
The c-di-GMP global pool modulates swimming motility using a multilevel regulation program. Swimming motility (A and C) and c-di-GMP concentrations (B and D) of all the DGC and PDE mutant intermediates were normalized to the wild-type EC1 levels. (A) Swimming motility decreased significantly to a first level after deleting *W909_14950* (bars 2 to 4) and then to an extremely lower level after deleting *W909_10355* (bars 5 to 8). The nonmotile Δ*fliG* strain was used as a negative control. (B) c-di-GMP concentrations of all consecutive PDE mutants showed reverse results with swimming motility. (C) The maximum swimming ability was shown after deleting *W909_14945* and remained unchanged despite deleting other DGC genes. (D) c-di-GMP concentrations were reduced extremely after deleting *W909_14945*, continuingly decreased after deleting *W909_02155*, and became undetectable after deleting *W909_06670*. Dotted lines indicate the wild-type levels of motility and c-di-GMP concentrations. Experiments were repeated three times in triplicates. Each sample was statistically compared to every other sample; the same lowercase letters indicate samples that are not significantly different, and different letters indicate significant differences (*P* < 0.0001 for panels A and C; *P* < 0.01 for panels B and D), as determined by one-way ANOVA with a multiple-comparison test.

Our construction of nested deletions in DGC- and PDE-encoding genes provided an opportunity to examine the effect of modulating c-di-GMP levels on some of the key processes that it controls. Thus, we analyzed the impact of DGC and PDE gene deletions on swimming motility, which was chosen as a key feature of virulence that can be easily quantified. Our analysis suggests that there are two PDEs that influence motility (Fig. 6A). Specifically, compared to the wild type, swimming was significantly decreased by about 50% in the *W909_14950* deletion mutant (1ΔPDE) and was further reduced upon the deletion of W909_10355 (EAL) (Fig. 6A). Similarly, the deletion of full-length *W909_10355* in the 3ΔPDE background showed results comparable to those for 4ΔPDE (Fig. S4). Furthermore, the double deletion of *W909_14950* and *W909_10355* resulted in motility comparable to that of 7ΔPDE (Fig. 6A). Interestingly, in contrast to the steadily changed patterns in the motility of PDE deletion mutants, bacteria showed the maximum swimming motility after deleting the first DGC gene, *W909_14945*, despite the consecutive deletion of other DGC genes (Fig. 6C). Moreover, both swimming motility and c-di-GMP concentrations of the key mutants 1ΔPDE, 4ΔPDE, and 1ΔDGC could be restored by in *trans* complementation by the target genes *W909_14950*, *W909_10355*, and *W909_14945*, respectively (Fig. S5).

10.1128/mBio.02993-20.4 FIG S4 Swimming motility and c-di-GMP levels of 4ΔPDE and 4ΔPDE(full) mutants. (A) Swimming motility. (B) c-di-GMP concentration. For easy comparison, the c-di-GMP concentration of each mutant was normalized to that of wild-type EC1, which was set to a value of 1. *, *P* < 0.0001; ns, *P* > 0.0001 (by Student’s *t* test). Error bars indicate standard deviations (*n* = 3 technical replicates). 4ΔPDE(full) represents the mutant strain with a deletion of the full length of *W909_10355* on the 3ΔPDE background. Download FIG S4, JPG file, 2.5 MB. Copyright © 2020 Chen et al. 2020 Chen et al. This content is distributed under the terms of the Creative Commons Attribution 4.0 International license.

10.1128/mBio.02993-20.5 FIG S5 Swimming motility and c-di-GMP levels of key mutants and complementary strains. For easy comparison, the swimming motility and c-di-GMP concentration of each mutant were normalized to those of wild-type EC1, which were set to a value of 1. Each sample was statistically compared to every other sample; the same lowercase letters indicate samples that are not significantly different, and different letters indicate significant differences (*P* < 0.0001), as determined by one-way ANOVA with a multiple-comparison test. Download FIG S5, TIF file, 0.9 MB. Copyright © 2020 Chen et al. 2020 Chen et al. This content is distributed under the terms of the Creative Commons Attribution 4.0 International license.

We then used liquid chromatography-mass spectrometry (LC-MS) to measure the c-di-GMP levels for all the deletion mutant strains. Mirroring the swimming phenotypes, the cellular c-di-GMP concentration was increased significantly after the deletion of *W909_14950* (1ΔPDE) (Fig. 6B) and showed a further increase after the deletion of the EAL domain-coding domain of *W909_10355* (4ΔPDE) (Fig. 6B). Conversely, consistent with swimming motility patterns (Fig. 6C), a sharp decrease in cellular c-di-GMP levels was observed upon the deletion of *W909_14945* (1ΔDGC) (Fig. 6D). The further deletion of *W909_02155* (2ΔDGC) reduced c-di-GMP levels by over 95%, and c-di-GMP became undetectable upon the deletion of *W909_02155* (4ΔDGC) (Fig. 6D).

By comparing the swimming motility and c-di-GMP concentration data, we could identify three levels of signal-function relationships. The first level occurred when c-di-GMP concentrations dropped by about 75%, with swimming motility increasing over 4-fold (Fig. 6C and D). The second level occurred when c-di-GMP increased approximately 2-fold, with swimming motility decreasing by about 2-fold (Fig. 6A and B). Finally, at the third level, the cells displayed the highest c-di-GMP levels (about 4-fold higher than those of the wild type) and were almost nonmotile (retained 10% of WT swimming).

**FIG 7:**
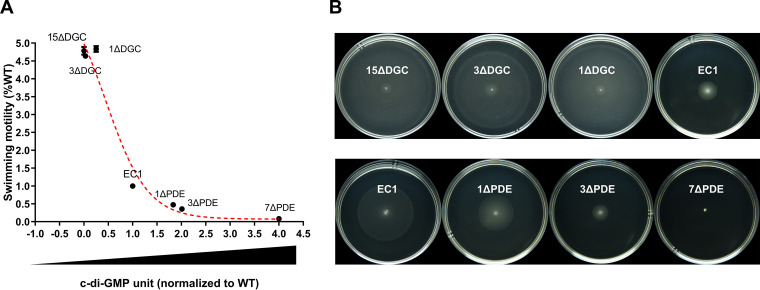
Swimming motility shows an L-shaped regression curve with c-di-GMP concentrations. (A) Swimming motility and c-di-GMP concentrations of 7 representative strains and mutants, as indicated, were chosen to fit the curve. (B) Swimming motility of the corresponding strains and mutants described above for panel A. Images were taken at 12 h (top row) and 24 h (bottom row). The experiment was repeated at least three times.

The above-described results suggest a relationship of the signal level to swimming motility that is not linear. Toward this point, we plotted the swimming activities of the wild type and the 6 mutant strains that produce varying levels of c-di-GMP (Fig. 7B). For the convenience of comparison, the c-di-GMP level and swimming activity of the wild type were arbitrarily set as 1. The results led to the establishment of an L-shaped regression curve (Fig. 7A), which depicts a clear relationship between the c-di-GMP level and swimming motility. Examination of this curve reveals that when the c-di-GMP level increases from 0 to 0.25 U, swimming motility is about 4-fold higher than that of the wild type. When c-di-GMP levels reach those of the wild type (1 U), swimming motility drops to 1. Finally, when c-di-GMP increased to levels that were either 1.83-, 2.01-, or 4-fold higher than those of the wild type, the bacterial swimming motility was reduced about 55% or 64% or the mutants became nonmotile, respectively.

### *D. zeae* EC1 c-di-GMP levels are primarily controlled by two PDE and one DGC enzyme.

**FIG 8:**
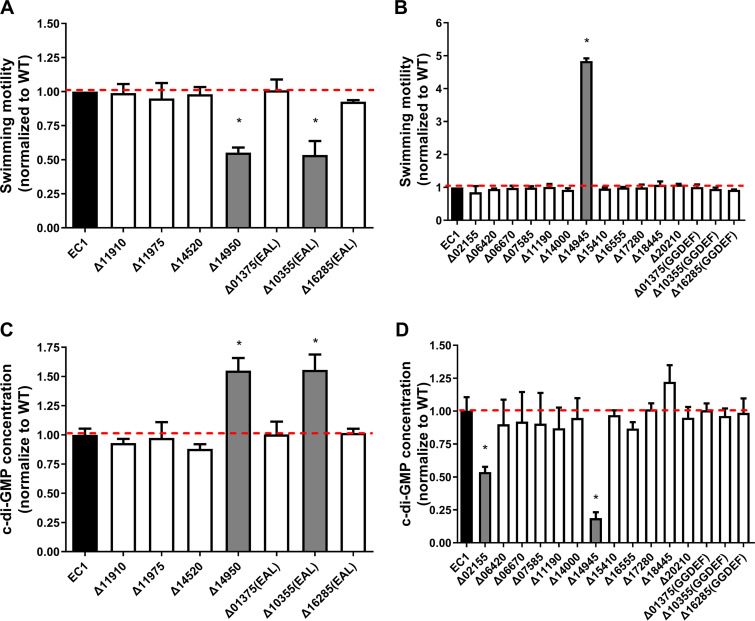
Swimming motility and c-di-GMP levels of individual PDE and DGC mutants. For easy comparison, the motility and c-di-GMP concentration of each mutant were normalized to those of wild-type EC1, which were set to a value of 1. *, *P* < 0.01 (Student’s *t* test). No “*” indicates that samples are not significantly different compared to EC1. Error bars indicate standard deviations (*n* = 3 technical replicates).

**FIG 9:**
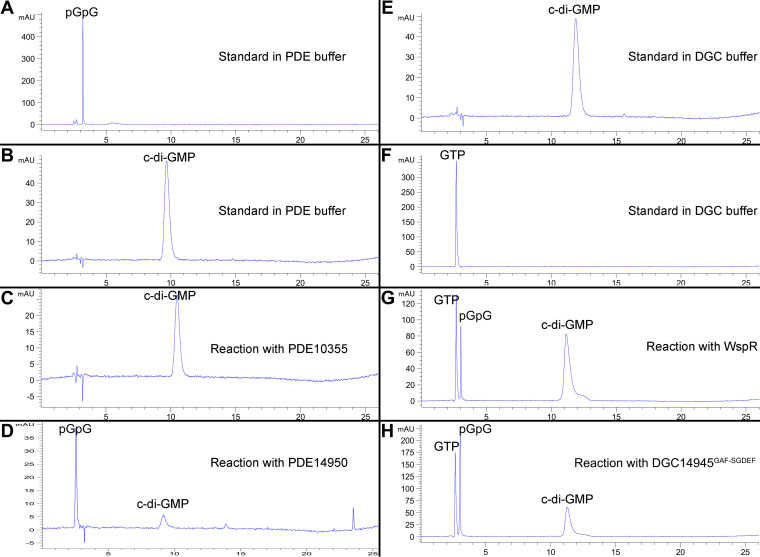
PDE and DGC activity assays of DGC14945, PDE14950, and PDE10355. (A to D) HPLC analysis of products in the c-di-GMP PDE enzymatic reaction. Purified PDE10355 and PDE14950 proteins were tested for PDE enzymatic activity using the c-di-GMP molecule as the substrate. (A) pGpG standard. (B) c-di-GMP standard. (C) When incubated with purified PDE10355 for 1 h, c-di-GMP was stable, and no degradation was detected. (D) When incubated with PDE14950 for 10 min, c-di-GMP was hydrolyzed into linear pGpG. (E to H) HPLC analysis of products in the c-di-GMP DGC enzymatic reaction. (E) c-di-GMP standard. (F) GTP standard. (G) GTP was synthesized by WspR into c-di-GMP after incubation for 60 min (positive control). (H) After incubation with purified DGC14945^GAF-SGDEF^ for 7 h, GTP was synthesized into c-di-GMP eventually. DGC14945, PDE14950, and PDE10355 represent *W909_14945*-, *W909_14950*-, and *W909_10355*-encoded proteins, respectively.

The results presented in Fig. 6 suggest that four genes, i.e., *W909_14950*, *W909_02155*, *W909_14945*, and *W909_10355*, might play a key role in modulating global levels of the c-di-GMP pool, hence affecting bacterial motility. Considering that the above-described findings were obtained by analysis of multiple deletion mutant strains, we decided to verify these findings by constructing and characterizing key single-gene deletions. The results showed that for the PDE-encoding genes, Δ10355(EAL) and Δ14950 showed significant decreases in swimming motility that corresponded to increases in c-di-GMP concentrations up to 1.5-fold compared to the wild type (Fig. 8A and C). For the individual deletions in the DGC-encoding genes, only one (Δ14945) was hypermotile with a corresponding drop in the c-di-GMP concentration (Fig. 8B and D). Interestingly, a mutation in the DGC-encoding gene Δ02155 produced approximately half as much c-di-GMP as the wild type but showed no significant changes in swimming motility (Fig. 8B and D), suggesting that the decrease in c-di-GMP biosynthesis in this mutant did not reach a level sufficient to influence motility. In addition, *W909_14945* and *W909_14950* encoding proteins DGC14945 and PDE14950, respectively, were purified, and their catalytic activities were confirmed by *in vitro* reverse-phase high-performance liquid chromatography (HPLC) assays (Fig. 9).

An obvious question relates to whether these genes are expressed under the culturing conditions used in this study. Given that certain media such as SOBS (SOB plus 1% sucrose) preferably promote biofilm formation (11), we decided to compare their transcript levels under different medium culture conditions, including minimal medium (MM), Luria-Bertani (LB) medium, SOBS, and swimming broth. The qRT-PCR analysis results showed that, in general, these c-di-GMP metabolic genes displayed comparable expression intensities in the 4 media (Fig. S6), suggesting that most of them are likely constitutively expressed under the culture conditions used in this study.

10.1128/mBio.02993-20.6 FIG S6 Expression profiles of the genes encoding c-di-GMP metabolism by qRT-PCR. Wild-type EC1 cells were inoculated under four conditions, i.e., MM, LB medium, motility medium without agar, and SOBS medium, at 28°C until the cells reached mid-log phase. Total RNA of each cell was extracted and reverse transcribed. M, reference gene *gapA*; 1, *W909_02155*; 2, *W909_06420*; 3, *W909_06670*; 4, *W909_07585*; 5, *W909_11190*; 6, *W909_14000*; 7, *W909_14945*; 8, *W909_15410*; 9, *W909_16555*; 10, *W909_17280*; 11, *W909_18445*; 12, *W909_21210*; 13, *W909_11910*; 14, *W909_11975*; 15, *W909_14520*; 16, *W909_14950*; 17, *W909_01375*; 18, *W909_10355*; 19, *W909_16285*. ns indicates a *P* value of >0.05 by Student’s *t* test. The experiment was repeated twice, and a representative set of data is shown. Download FIG S6, TIF file, 2.0 MB. Copyright © 2020 Chen et al. 2020 Chen et al. This content is distributed under the terms of the Creative Commons Attribution 4.0 International license.

### High c-di-GMP levels inhibit motility through a PilZ domain protein.

**FIG 10:**
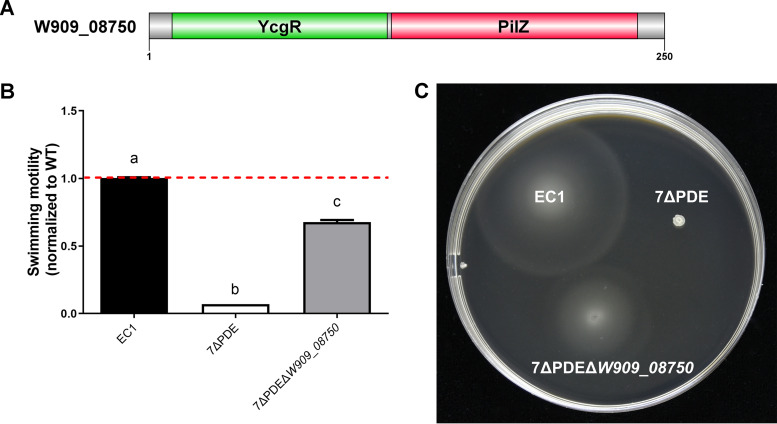
Domain structure of W909_08750 and swimming motility of EC1 and its derivatives. (A) Predicted domain architectures of W909_08750. Domains of the proteins were predicted and represented using DOG 2.0 software (67). YcgR is involved in motility control in enterobacteria. PilZ is a receptor domain specific for c-di-GMP (68). (B and C) Swimming motility of EC1, 7ΔPDE, and 7ΔPDEΔ*W909_08750*. Cultures grown overnight were adjusted to the same cell density (OD_600_ = 2.0), and 1 μl was inoculated onto swimming agar plates. Data were collected after 18 h at 28°C. Dotted lines indicate the wild-type level of swimming motility. Experiments were repeated three times in triplicates. Each sample was statistically compared to every other sample; the same lowercase letters indicate samples that are not significantly different, and different letters indicate significant differences (*P* < 0.0001), as determined by one-way ANOVA with a multiple-comparison test.

PilZ-containing proteins were identified as the most important and ubiquitous c-di-GMP-specific binding receptors over 10 years ago through comparative genome analysis and then confirmed by chemistry experiments *in vivo* (40, 41). Using position-specific iterated BLAST analysis, a PilZ domain-containing protein, W909_08750, was identified in the *D. zeae* EC1 genome, which contains the PilZ domain in the C terminus and a YcgR-homologous domain in the N terminus (Fig. 10A). YcgR is the flagellar brake protein in Escherichia coli that directly binds to c-di-GMP to regulate swimming motility in that species. Mutation of *W909_08750* in the 7ΔPDE background could partially restore cell motility compared to wild-type EC1 (Fig. 10B and C), which is consistent with previous findings in E. coli (42).

## DISCUSSION

In this study, we investigated the impact of c-di-GMP signaling on *D. zeae* physiology and virulence. To this end, we generated a c-di-GMP-null (cdG^0^) mutant strain by deleting all 15 genes with potential c-di-GMP cyclase activity. This strain showed increased flagellum-mediated motility (Fig. 2; see also Fig. S1 in the supplemental material) and an abolished biofilm-forming capacity (Fig. 2C). However, virulence on rice seeds was not affected (Fig. 2D and E). These findings are in contrast to those of previous studies in *Caulobacter*, for which the cdG^0^ strain displayed severely abolished motility (29). Our results also differed from those of a similar study performed in S. meliloti and *Salmonella*, where a cdG^0^ strain exhibited comparable effects on biofilm formation and motility compared to wild-type strains, respectively (30, 31). However, the cdG^0^ strain appeared to show a reduced ability to inhibit germination at low dilution levels (Fig. 2D), which might suggest an impact of quorum sensing on virulence. Transcriptional analysis revealed that the cdG^0^ strain had elevated mRNA levels of flagellar genes (Fig. 3). In contrast, the cdG^0^ mutant strain of *Caulobacter* could not assemble flagella (29).

A c-di-GMP signaling mutant background containing mutations in all 7 genes with predicted PDE activity was also constructed. This strain, complementary to the cdG^0^ strain, gave us some insight into c-di-GMP signaling in *D. zeae*. This strain showed no swimming motility, formed extensive biofilms, and could not invade rice seeds (Fig. 4 and Fig. S1). Further analysis showed that this strain exhibited reduced transcript levels for the genes associated with flagellum biosynthesis (Fig. 5). This is in contrast to the *Enterobacteriaceae*, where elevated c-di-GMP levels in E. coli and Salmonella enterica serovar Typhimurium inhibit motility by the suppression of flagellar rotation rather than flagellar biosynthesis (31, 42, 43). In *Pseudomonas* and *Vibrio* spp., c-di-GMP binds to the master flagellar regulator FleQ/FlrA and negatively regulates the expression of flagellar genes (36, 44, 45), whereas *D. zeae* EC1 belongs to enteric bacteria, which has no protein homologous to FleQ/FlrA and possesses FlhDC as the master flagellar regulator to control flagellar gene expression. As the regulation of flagella by c-di-GMP is central in this study, information on the effect on flagellar gene transcription levels, especially on the master regulator *flhDC*, is probably important, which remains to be further investigated.

Previous studies showed that a mutant strain unable to produce the phytotoxin zeamine in *D. zeae* EC1 was unable to inhibit rice seed germination, suggesting that zeamines are key virulence factors (13). In contrast, we found that the PDE-null strain did not affect zeamine production (Fig. S2) but inhibited motility and attenuated virulence on rice seeds (Fig. 4). This is similar to our recent findings in which blocking putrescine signaling did not affect zeamine production in *D. zeae* EC1 but led to decreased bacterial swimming motility and an attenuated invasion capability (18). We also found that the nonmotile Δ*fliG* mutant significantly decreased the colonization and inhibition ability, which was comparable to the 7PDE strain (Fig. S3). However, the biofilm formation defect of the Δ*bcsA* mutant strain seemed to lead to an invasion defect (Fig. S3E), although its GFP intensity was not significantly decreased (Fig. S3A), which is similar to the finding in D. dadantii that a single mutation of the *bcsA* gene could not attenuate virulence, whereas it decreased the bacterial surface colonization ability (46). The above-mentioned lines of evidence highlight that motility is an important aspect of virulence (47), while zeamine can effectively target the rice stem and roots, which leads to inhibition of seed germination. The above-mentioned notion perhaps helps explain the puzzling observations that while a low inoculum of strain EC1 could provide total inhibition of rice seed germination (17), the addition of 480 μM purified zeamines, which is equivalent to the amount of zeamines produced by 5 liters of an EC1 culture, could only partially inhibit germination (13). Moreover, there may be more reasons for the attenuation of virulence via the c-di-GMP signaling network, which was reported to regulate numerous aspects of cell physiology in bacteria (48).

This study identified a few primary c-di-GMP signaling genes that appear to carry out the bulk of c-di-GMP synthesis or degradation in laboratory culture. Although strain EC1 encodes multiple DGC and PDE enzymes, our results showed that only a few played a decisive role in influencing c-di-GMP levels and the associated phenotypes in four different medium types. These genes included the c-di-GMP cyclase DGC14945 and the phosphodiesterases PDE10355 and PDE14950 (Fig. 6 and 9). Although we detected the PDE enzymatic activity of PDE10355 only *in vivo*, it contains both canonical GGDEF and EAL motifs, which are predicted to be active. In Agrobacterium tumefaciens and other proteobacteria, the dual DGC-PDE function of the DcpA protein was controlled by pterins (49). This could also be the situation for PDE10355 in *D. zeae*, which remains to be determined. It is intriguing that among the 19 predicted c-di-GMP signaling proteins, only a few appeared to influence c-di-GMP pools (Fig. 8). Protein sequence alignment showed that among all six EAL motif-harboring proteins (see Fig. S7 in the supplemental material), three of them (W909_14950, W909_11910, and W909_01375) lack at least one essential key motif (Fig. S7, red arrows) (37); however, W909_14950 showed a high score of homology with YhjH, a PDE protein in E. coli (50). The other PDE proteins, i.e., W909_10355, W909_11975, and W909_16285, contain the canonical motif (51). Alignment of the amino acids of all 15 GGDEF domain-containing proteins (see Fig. S8 in the supplemental material) showed that only 3 of them (W909_11190, W909_14945, and W909_01375) diverge from the canonical GG(D/E)EF motif (Fig. S8, red arrows). The first glycine of the GG(D/E)EF motif in W909_11190 and W909_14945 was systematically replaced with serine and alanine, respectively, while W909_14945 displayed the same SGDEF active-site motif with a noncanonical diguanylate cyclase, ECA3270, in Pectobacterium atrosepticum (52), and we have confirmed that W909_14945 is a protein with DGC activity both *in vivo* and *in vitro* (Fig. 8 and 9). However, overexpressing *W909_14945* in 1ΔDGC could only partially complement this strain (Fig. S5). Therefore, it could have additional functionality to regulate motility since it possesses domains other than SGDEF, namely, PAC, PAS, and GAF domains. This remains to be further investigated. The third noncanonical DGC, W909_01375, lost the whole GG(D/E)EF motif, having YHSDF in place of the usual motif (Fig. S8). These observations led to the natural question of whether these genes are all being expressed under the laboratory culturing conditions. Interestingly, qRT-PCR analysis suggested that all 19 of the putative c-di-GMP signaling genes in strain EC1 are transcribed and maintain similar transcript profiles under the 4 culture conditions tested (Fig. S6). This of course does not confirm that stable proteins are being expressed. In addition, other explanations for the lack of activity could be either that there is a nonfunctional catalytic motif (37) or, in some cases, enzyme activity requires a signal that is missing under the conditions tested (53).

10.1128/mBio.02993-20.7 FIG S7 Sequence alignment of the EAL domains with conserved residues. Conserved residues that form the phosphodiesterase activity of the EAL domain are shown by red and black backgrounds, which indicate ≥75% and 100% homology, respectively. Essential residues are indicated by a black asterisk. Positive controls are RocR (UniProt accession no. Q9HX69) from P. aeruginosa PAO1, HmsP (UniProt accession no. A0A384L250) from Yersinia pestis, and YhjH (UniProt accession no. P37646) from E. coli. Download FIG S7, TIF file, 2.2 MB. Copyright © 2020 Chen et al. 2020 Chen et al. This content is distributed under the terms of the Creative Commons Attribution 4.0 International license.

10.1128/mBio.02993-20.8 FIG S8 Sequence alignment of the GGDEF domains with conserved residues. Conserved residues that form the diguanylate cyclase activity of the GGDEF domain are shown in white on a red or black background. The essential residues (GGDEF motif) are indicated by black asterisks. Positive controls are PleD (UniProt accession no. B8GZM2) from C. crescentus, WspR (UniProt accession no. Q9HXT9) from P. aeruginosa PAO1, and HmsT (UniProt accession no. Q9X5X6) from Yersinia pestis. Download FIG S8, JPG file, 2.6 MB. Copyright © 2020 Chen et al. 2020 Chen et al. This content is distributed under the terms of the Creative Commons Attribution 4.0 International license.

Our findings also provide insight into the dose-function relationship of c-di-GMP and motility for *D. zeae*. Our results with *D. zeae* indicate that the swimming motility follows an L-shaped regression curve with c-di-GMP levels (Fig. 7A), which is distinct from the C. crescentus findings where motility and surface attachment followed an inverted U-shaped c-di-GMP dose-response curve (29). In this study, we measured motility over a range of c-di-GMP conditions in select mutant backgrounds. It may be possible that this difference is due to the different needs of these two bacterial species for c-di-GMP in motility regulation. To be specific, C. crescentus needs a relatively low level of c-di-GMP to initiate flagellum assembly and obstruct motility when c-di-GMP reaches a threshold (29), while *D. zeae* limits motility once c-di-GMP levels start to rise (Fig. 6).

Furthermore, several regulatory mechanisms associated with c-di-GMP turnover have been implicated in *D. zeae* virulence from our previous findings; specifically, the two-component system proteins VfmIH and a global transcriptional regulator, SlyA, were reported to regulate some key c-di-GMP turnover enzymes at the transcriptional level, including the primary DGC14945 enzyme (14, 16). The AHL quorum sensing signal as well as host-pathogen communication through putrescine signaling were reported to mainly regulate flagellar motility and biofilm formation (17, 18). Given that the overlap between c-di-GMP and other regulatory systems is widespread within numerous bacterial species, it would be interesting to investigate the connection between c-di-GMP and these systems.

Taken together, these findings present new insight into the roles and molecular mechanisms of c-di-GMP genes in the regulation of bacterial physiology and virulence. We showed that the cdG^0^ mutant became highly motile but maintained virulence similar to that of wild-type EC1. We also found that the PDE-free mutant was attenuated in virulence mainly due to the abrogation of cell motility. Furthermore, this study identified three key genes playing critical roles in modulating the c-di-GMP levels in bacterial cells and that c-di-GMP regulated bacterial motility following an L-shaped regression curve. These features are different from or dissimilar to those identified in other bacterial species such as *Salmonella* and *Caulobacter* (29, 31), suggesting the complexity and plasticity of c-di-GMP regulatory circuits in different bacterial species.

## MATERIALS AND METHODS

### Bacterial strains and plasmids.

Bacterial strains and plasmids used in this study are described in Table S1 in the supplemental material. Escherichia coli was routinely grown at 37°C in Luria-Bertani (LB) medium. *D. zeae* EC1 and its derivatives were grown at 28°C in LB medium as previously reported. Minimal medium (MM) agar plates were used for conjugation (17). For measuring zeamine production, bacteria were grown in LS5 medium as previously described (19). For assays of biofilm formation, strains were grown in SOBS medium, which consists of SOB plus 1% sucrose (containing 20 g tryptone, 5 g yeast extract, 2.4 g MgSO_4_, 0.5 g NaCl, 0.186 g KCl, and 10 g sucrose per liter) (11). The following antibiotics were added at the indicated final concentrations when required: ampicillin (Ap) at 100 μg/ml, kanamycin (Km) at 50 μg/ml, streptomycin (Str) at 50 μg/ml, and polymyxin (Pm) at 30 μg/ml. The optical density at 600 nm (OD_600_) of the bacterial culture was measured by using a NanoDrop 2000c system (Thermo Fisher Scientific, USA) at 600 nm.

10.1128/mBio.02993-20.9 TABLE S1 Strains and plasmids used in this study. Download Table S1, PDF file, 2.2 MB. Copyright © 2020 Chen et al. 2020 Chen et al. This content is distributed under the terms of the Creative Commons Attribution 4.0 International license.

### Mutant construction and complementation.

The generation of in-frame gene deletion mutants was conducted using the suicide vector pKNG101 and triparental mating, according to a protocol described previously (19). In-frame deletion of the coding regions of the specific GGDEF or EAL domain of three genes, i.e., *W909_01375*, *W909_10355*, and *W909_16285*, was done by the allelic-exchange method (54). Flanking regions of each coding region were amplified by PCR using the specific primers listed in Table S2 in the supplemental material. A complementation assay was conducted by using the plasmid pBBR1MCS-4 and triparental mating, according to a protocol described previously (19). The coding region of the target gene was amplified by PCR using specific primers (Table S2).

10.1128/mBio.02993-20.10 TABLE S2 Primers used in this study. Download Table S2, PDF file, 0.07 MB. Copyright © 2020 Chen et al. 2020 Chen et al. This content is distributed under the terms of the Creative Commons Attribution 4.0 International license.

### Extracellular enzyme activity assays.

The cellulase (Cel), pectate lyase (Pel and Peh), and proteolytic enzyme (Prt) activities were measured using carboxymethyl cellulose sodium, polygalacturonic acid, and skimmed milk as the substrates, respectively, according to methods and conditions described previously (19).

### Zeamine production assay.

A zeamine production assay was carried out in LS5 medium as previously described (19).

### Flagellum-mediated motility assay.

Collective swimming motility was assessed in a semisolid medium plate with 0.2% agar (each liter containing 10 g Bacto tryptone, 5 g NaCl, and 2 g agar). A bacterial culture grown overnight (1 μl) was spotted on the center of the plate and incubated at 28°C for 12 to 24 h before measurement. Collective swarming motility was assayed as previously described (19).

### Biofilm formation assay.

A biofilm formation assay was performed according to the procedure described previously by Kulasakara et al. (55), with minor modifications. A bacterial culture grown overnight was diluted 1:1,000 in SOBS medium, and 2 ml was then transferred to 10-ml glass tubes and incubated at 28°C for 24 h without shaking. The bacterial biofilm mass was stained with 2 ml 0.1% (wt/vol) crystal violet for 15 min after pouring off the medium gently and washing with water at least three times, and tubes were rinsed with water three times until all unbound dye was removed. For measuring the biofilm mass, stained cells in each tube after dryness were decolorized with 3 ml of 70% ethanol and quantified by the absorbance at 570 nm. Three independent assays were carried out for each bacterial strain.

### Rice seed germination assay.

A rice seed germination assay was performed according to a previously described method, with minor modifications (17). Briefly, bacterial cultures grown overnight were diluted (1:100) in LB medium and cultured at 28°C with shaking at 200 rpm. Supernatants were removed after centrifugation at 4,000 rpm for 10 min; cells were resuspended in 1× phosphate-buffered saline (PBS) buffer (pH 7.4), adjusted to an OD_600_ of 0.6, and then diluted in 8-fold series with 1× PBS buffer; and the CFU of the last three dilution series were measured using a heterotrophic plate counting assay. Fifteen rice seeds of cultivar CO39 were immersed in 10 ml of bacterial dilution cultures of each strain for 6 h at room temperature, washed with sterilized water three times, and transferred onto two Whatman paper no. 3 filter papers moistened with sterilized water in a petri dish. The conditions for rice seed germination were set at 28°C under 16 h of light and 8 h of dark for 1 week, adding sterilized water when necessary. The same amount of 1× PBS buffer was used as a blank control. The germination inhibition ratio was measured at the time points indicated, and the experiment was repeated three times with triplicates.

### Microscopy visualization of rice seed colonization.

Bacterial cultures of *D. zeae* strain EC1 and its derivatives carrying the pLAFR3-GFP plasmid were grown in LB medium overnight. Bacterial cells were collected by centrifugation at 4,000 rpm for 10 min and resuspended in PBS buffer until the CFU reached 1 × 10^5^. Fifteen rice seeds of rice variety CO39 were immersed in 10 ml of bacterial dilutions of each strain for 6 h, washed with sterilized water three times, and transferred onto two moistened Whatman paper no. 3 filter papers in a petri dish. The seeds were placed at 28°C under a 16-h-light and 8-h-dark cycle and harvested after 40 h. Each seed with the husk removed was placed on a microscope slide without a coverslip and visualized using a Leica DMi8 fluorescence inverted microscope (Leica, Wetzlar, Germany) at a ×5 magnification. The fluorescence images were taken using fluorescence filters (for GFP, excitation [EX] at 450 to 500 nm, emission [EM] at 512 to 542 nm, and dichroic mirror [DC] at 505 nm; for seed spontaneous red fluorescent protein [RFP] fluorescence, EX at 540 to 580 nm, EM at 592 to 668 nm, and DC at 585 nm). The gain factor for fluorescence was set manually to 3.0 with a 600-ms exposure time, and green and red fluorescence photographs were merged using Leica LAS-X software (Leica, Wetzlar, Germany). According to the autofluorescence properties of plant cells, the RFP channel was used to visualize the profile of the rice seed, which distinguished plant tissues from the bacterial cells expressing GFP fluorescence (56). The experiment was repeated three times with five seeds in each treatment or control group. The fluorescence intensity in the regions of interest (ROIs) in the GFP channel of at least 5 representative samples was measured using ImageJ software (W. Rasband, National Institutes of Health, USA).

### Transmission electron microscopy for examination of flagella.

Bacterial cells were cultured in LB medium until mid-log phase and then adsorbed onto glow-discharged, carbon-coated colloid film on a copper grid. The grids were then washed three times with deionized water and negatively stained with 2% (wt/vol) phosphotungstic acid for 2 min. Specimens were examined with an FEI Talos F200 microscope at 200 kV. The experiment was repeated twice, each time with over 100 bacterial cells per sample being examined. At least 20 cells of each strain were taken to calculate the number of flagella.

### Quantitative analysis of c-di-GMP by LC-MS.

Quantification of c-di-GMP levels was performed based on a protocol described previously by Hickman and Harwood (45), with modifications. Cells were grown overnight in LB medium, adjusted to an OD_600_ of 2.0, and then subcultured in 4 ml minimal medium with a 50-fold dilution in a 14-ml culture tube (Crystalgen, USA). When the bacterial culture was grown to an OD_600_ of ∼0.4 to 0.6, an aliquot of 1 ml was transferred into a 2-ml microcentrifuge tube, and perchloric acid (70%, vol/vol) was added to a final concentration of 0.6 M. For bacterial mutants producing low levels of c-di-GMP, 10 to 20 ml of a fresh bacterial culture (OD_600_ = ∼0.4 to 0.6) was condensed by centrifugation and suspended in 1 ml minimal medium before perchloric acid dissolution. The samples were placed in ice for at least 30 min. Next, cell debris was collected by centrifugation at 4°C for 10 min at 12,000 rpm and used for subsequent protein determination. Supernatants were transferred into a 15-ml conical tube and neutralized by adding a 1/5 volume (about 219 μl) of 2.5 M KHCO_3_. The resulting salt precipitates were removed by centrifugation at 4°C for 10 min at 4,000 rpm using a 5810 R fixed-angle rotor centrifuge (Eppendorf, Germany). Supernatants were transferred into a 2-ml microcentrifuge tube and stored at −80°C before c-di-GMP analysis by LC-MS.

Five microliters of each sample was analyzed using a Q Exactive Focus hybrid quadrupole-orbitrap mass spectrometer (Thermo Fisher Scientific, USA). Cyclic di-GMP separations were achieved using a 100- by 2.1-mm Syncronis C_18_ column (Thermo Fisher Scientific, USA). A gradient system was operated with an isocratic elution protocol with 95% aqueous (2.5 mM ammonium acetate) and 5% organic (methanol) phases. The flow rate was 0.2 ml min^−1^, and the cycle time was 10 min. c-di-GMP was detected with an orbitrap mass analyzer on the Q Exactive Focus system (Thermo Fisher Scientific, USA) in positive ionization mode. The ion spray voltage was 3.5 kV, and sheath gas and auxiliary gas flow rates were 45 and 10 units, respectively. A mass-to-charge ratio (*m/z*) of 691.10214 was used as a confirmatory signal, and c-di-GMP quantification was performed in selected ion monitoring (SIM) mode with a resolution and an automatic gain control (AGC) target of 35,000 and 5e4, respectively.

For a standard curve, 2.5, 5, 10, 20, 50, 100, and 500 nM pure c-di-GMP (Biolog, Germany) were analyzed by the method described above. c-di-GMP levels were normalized to the total protein per milliliter of culture. Data represent the means from three independent cultures, with error bars indicating the standard deviations.

For protein quantitation, precipitated fractions were resuspended in 100 μl 1 M NaOH and heated in an ion bath at 100°C for 10 min. Samples were then cooled to room temperature, and a protein assay was carried out using a Coomassie Plus (Bradford) assay kit (Thermo Fisher Scientific, USA). Bovine serum albumin (BSA) was used as a standard. The experiment was repeated twice with triplicates.

### Protein expression and purification.

The coding regions corresponding to full-length PDE10355 (*W909_10355*) and PDE14950 (*W909_14950*) were amplified by PCR using EC1 genomic DNA as the template and were respectively cloned into the pET-28b(+) plasmid at the NcoI and XhoI sites using a ClonExpress II one-step cloning kit (Vazyme Biotech Co., Ltd., China). The coding sequence of DGC14945^GAF-SGDEF^ (residues 174 to 504 of *W909_14945*) was amplified by PCR using EC1 genomic DNA as the template and then cloned into the pET-32a(+) expression plasmid at the BamHI and HindIII sites using the same cloning kit. Recombinant plasmids were respectively transformed into E. coli DH5α competent cells for sequencing, and the correct constructs of pET-PDE10355, pET-DGC14945^GAF-SGDEF^, and pET-PDE14950 were respectively transformed into E. coli BL21(DE3) for fusion protein expression. For the overexpression of fusion proteins, cultures of the expression strains grown overnight were inoculated into 200 ml LB medium in 500-ml Fernbach flasks and grown with shaking until the OD_600_ reached ∼0.4 to 0.6, isopropyl-β-d-thiogalactopyranoside (IPTG) was added to a final concentration of 0.5 mM to induce the expression of fusion proteins, and cultures were placed in a shaker at 15°C and grown by shaking overnight. Cells were harvested by centrifugation at 4,000 rpm for 15 min at 4°C and resuspended in 20 ml xTractor buffer (Clontech, TaKaRa Biomedical Technology [Beijing] Co., Ltd., China) for lysis. Cell suspensions were incubated at room temperature for 10 min with gentle shaking. Crude lysates were centrifuged at 4,000 rpm for 20 min at 4°C, and supernatants were filtrated using 0.45-μm syringe filters (Pall Corporation). Affinity purification was performed at 4°C using Talon metal affinity resins (Clontech, TaKaRa Biomedical Technology [Beijing] Co., Ltd., China). The resin and the column were washed with 10 column volumes of equilibration buffer (50 mM sodium phosphate, 300 mM sodium chloride, 20 mM imidazole [pH 7.4]) before adding the supernatant lysate. The target protein was eluted with gradient elution buffer (50 mM sodium phosphate, 300 mM sodium chloride, 50/100/150/200/300 mM imidazole [pH 7.4]). All the fractions were estimated by Coomassie brilliant blue staining after SDS-PAGE. The protein concentration was determined at 280 nm using the NanoDrop 2000c system (Thermo Fisher Scientific, USA).

### Enzyme activity assay and high-performance liquid chromatography analysis.

PDE enzyme activity was measured according to a procedure described previously by Yi et al., with modifications (57). The reaction mixture for PDE activity contained 4 μg protein and 50 μM c-di-GMP in 1 ml reaction buffer (50 mM Tris-HCl [pH 7.6], 10 mM MgCl_2_, 50 mM NaCl). The mixture was incubated in an ion bath at 37°C for 1 h, and 100 μl of each sample was taken out every 10 min and stopped by adding 10 μl of 1 M CaCl_2_ and placing the sample in a 95°C ion bath for 5 min.

The DGC reaction mixture contained 50 μg of protein, 500 μM GTP, 75 mM Tris-HCl (pH 7.8), 250 mM NaCl, 25 mM KCl, and 10 mM MgCl_2_ in a total volume of 500 μl. The mixture was incubated at 37°C for 7 h, and 100 μl of each sample was taken out at different time points as indicated and stopped by placing the sample in a 95°C ion bath for 5 min. The above-described reaction products were centrifuged at 12,000 rpm for 2 min, and supernatants were analyzed by reverse-phase high-performance liquid chromatography (HPLC), according to the same method developed previously by Chua et al. (58). Ten microliters of each sample was injected into an Agilent 5 HC-C_18_(2) 250- by 4.6-mm column (Agilent, CA, USA) using a flow rate of 1 ml/min with the same solvents and elution gradient as the ones previously reported by Chua et al. (58). Nucleotides were detected at a wavelength of 254 nm.

### RNA isolation, cDNA synthesis, and qRT-PCR analysis.

Bacterial cells grown in LB medium (OD_600_ = 0.6) were harvested, and total RNAs were isolated using TRIzol reagent and the Phasemaker Tubes complete system (Invitrogen, Thermo Fisher Scientific, USA) according to the manufacturer’s protocol. RNA quality and integrity assessment were performed using the NanoDrop 2000c system (Thermo Fisher Scientific, USA) and agarose gel electrophoresis.

For cDNA synthesis, 1 μg total RNA of each sample was used as the template to synthesize cDNA using the FastQuant RT kit (with genomic DNase) (Tiangen Biotech, China). Talent qPCR PreMix (SYBR green) (Tiangen Biotech, China) was used for qRT-PCR to quantify the transcript levels of the c-di-GMP turnover gene, with *gapA* or *recA* used as the internal reference gene according to MIQE guidelines (59, 60). Primers for SYBR green qRT-PCR were designed using the Beacon designer (Premier Biosoft) and are listed in Table S2. Assays were performed in quadruplicate in 10-μl reaction mixtures using the Applied Biosystems QuantStudio 6 Flex real-time PCR system (Thermo Fisher Scientific, USA). PCR was performed according to the manufacturer’s instructions. The gene expression level was analyzed and calculated using ΔΔ*C_T_* methods as previously described (61). Data represent the means from three independent biological repeats, with error bars indicating the standard deviations.

### Bacterial growth analysis.

Bacterial cultures grown overnight in LB broth were diluted (1:100) in the same medium and adjusted to the same cell density. Two hundred microliters of the diluted culture was grown in each well at 28°C in a low-intensity shaking model using the Bioscreen-C automated growth curve analysis system (OY Growth Curves AB, Ltd., Finland).

### Data availability.

The genome sequence of *D. zeae* EC1 is accessible in the NCBI database under accession no. NZ_CP006929.1. The nucleotide sequences of *wspR* and *rocR* in P. aeruginosa PAO1 are accessible in the NCBI database under gene IDs 878337 and 878871.
