# Supplementary figures and images for: Systematic Analysis of c-di-GMP Signaling Mechanisms and Biological Functions in Dickeya zeae EC1
Source: mBio. 2020 Dec 1;11(6):e02993-20. doi: 10.1128/mBio.02993-20 (PMC7733949; doi:10.1128/mBio.02993-20)

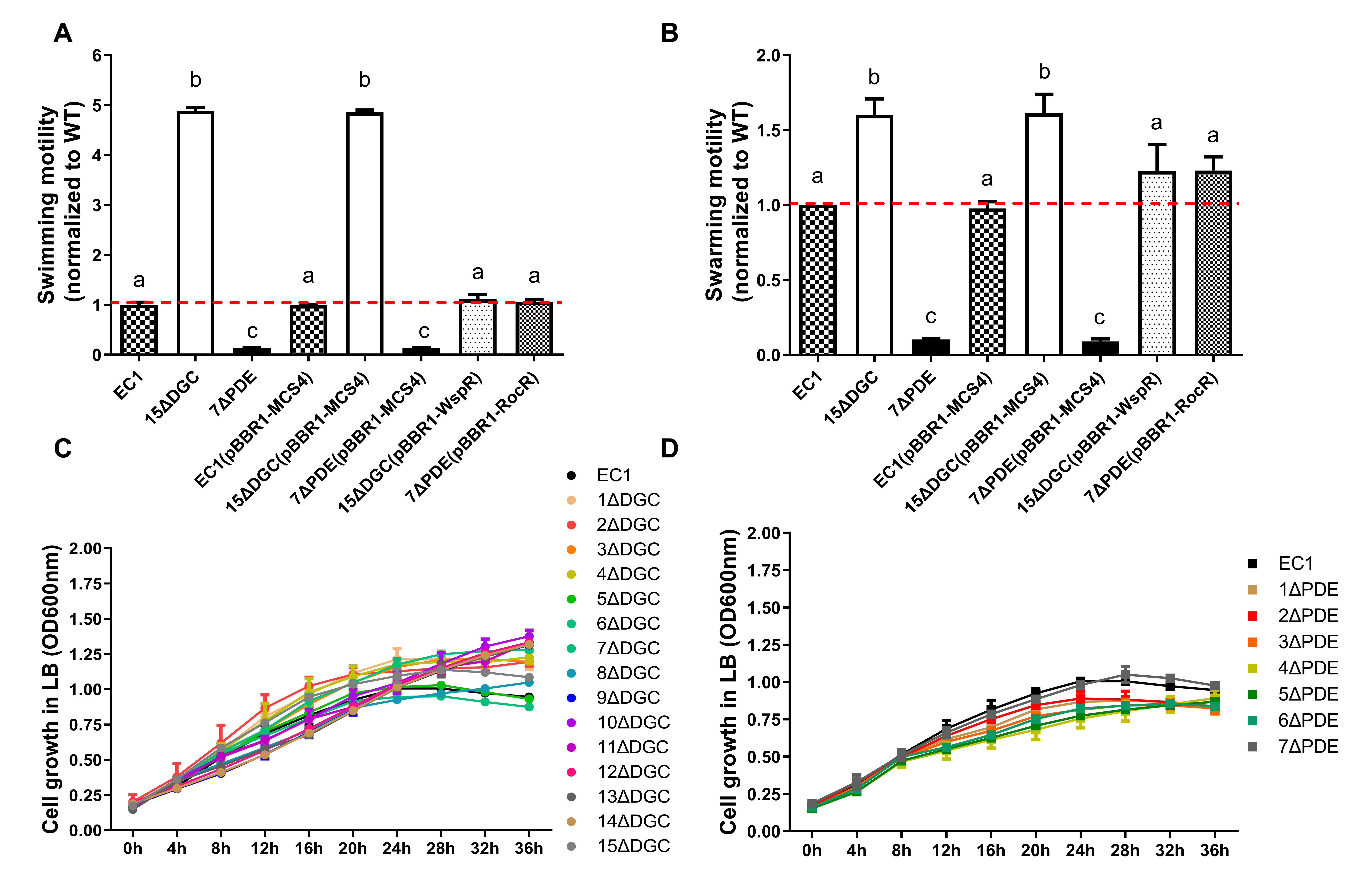

Supplement: FIG S1 [file mBio.02993-20-sf001.tif]

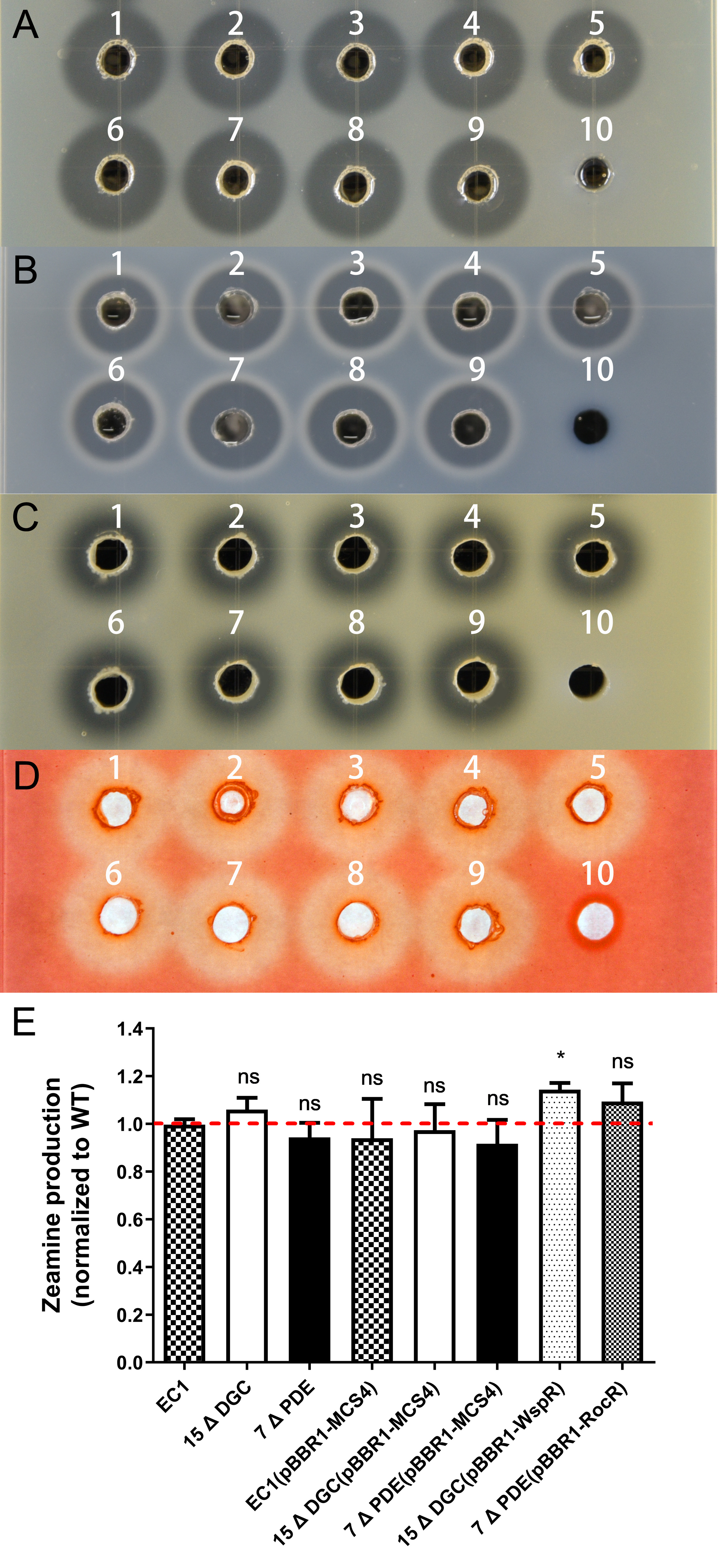

Supplement: FIG S2 [file mBio.02993-20-sf002.jpg]

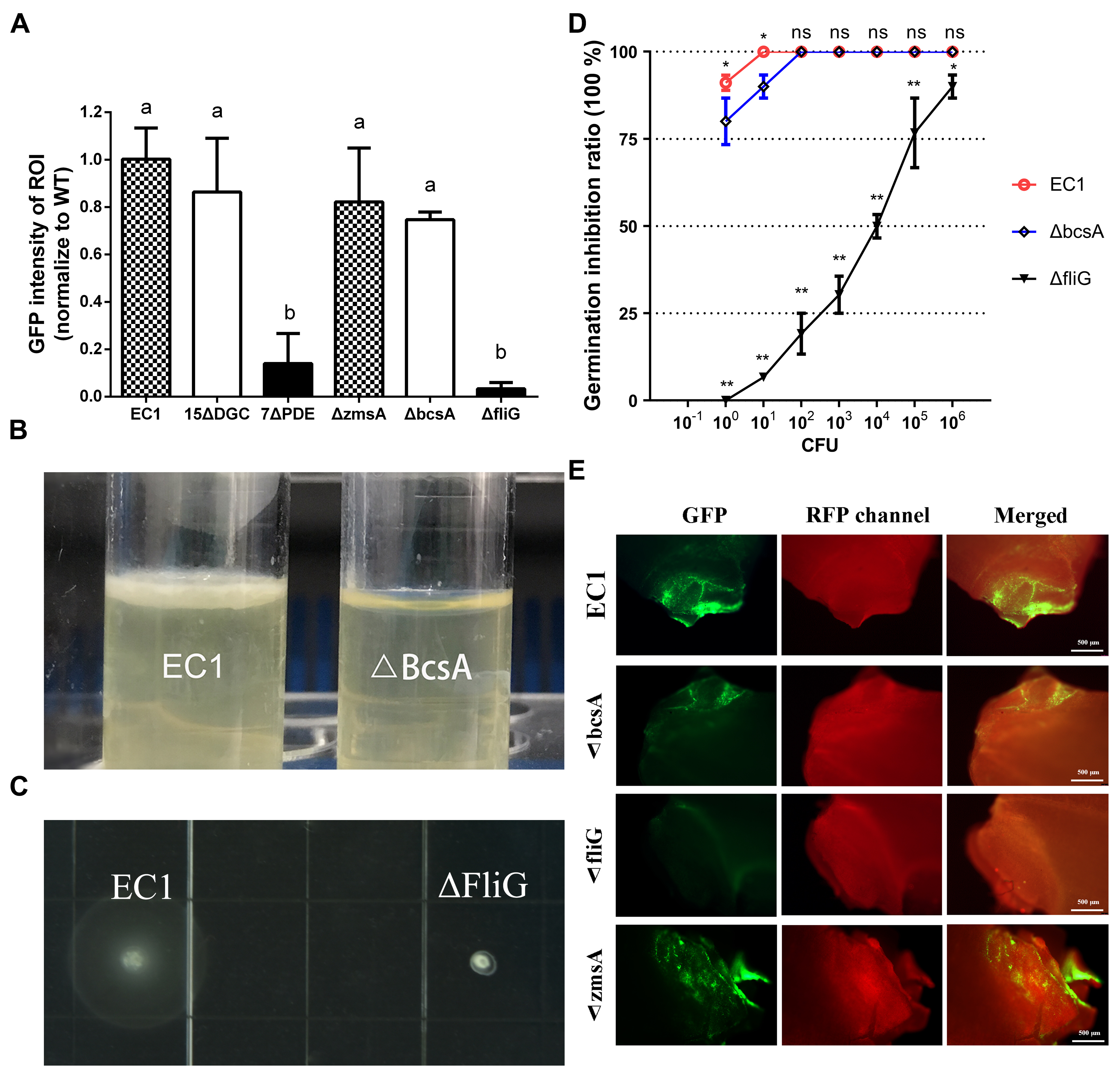

Supplement: FIG S3 [file mBio.02993-20-sf003.jpg]

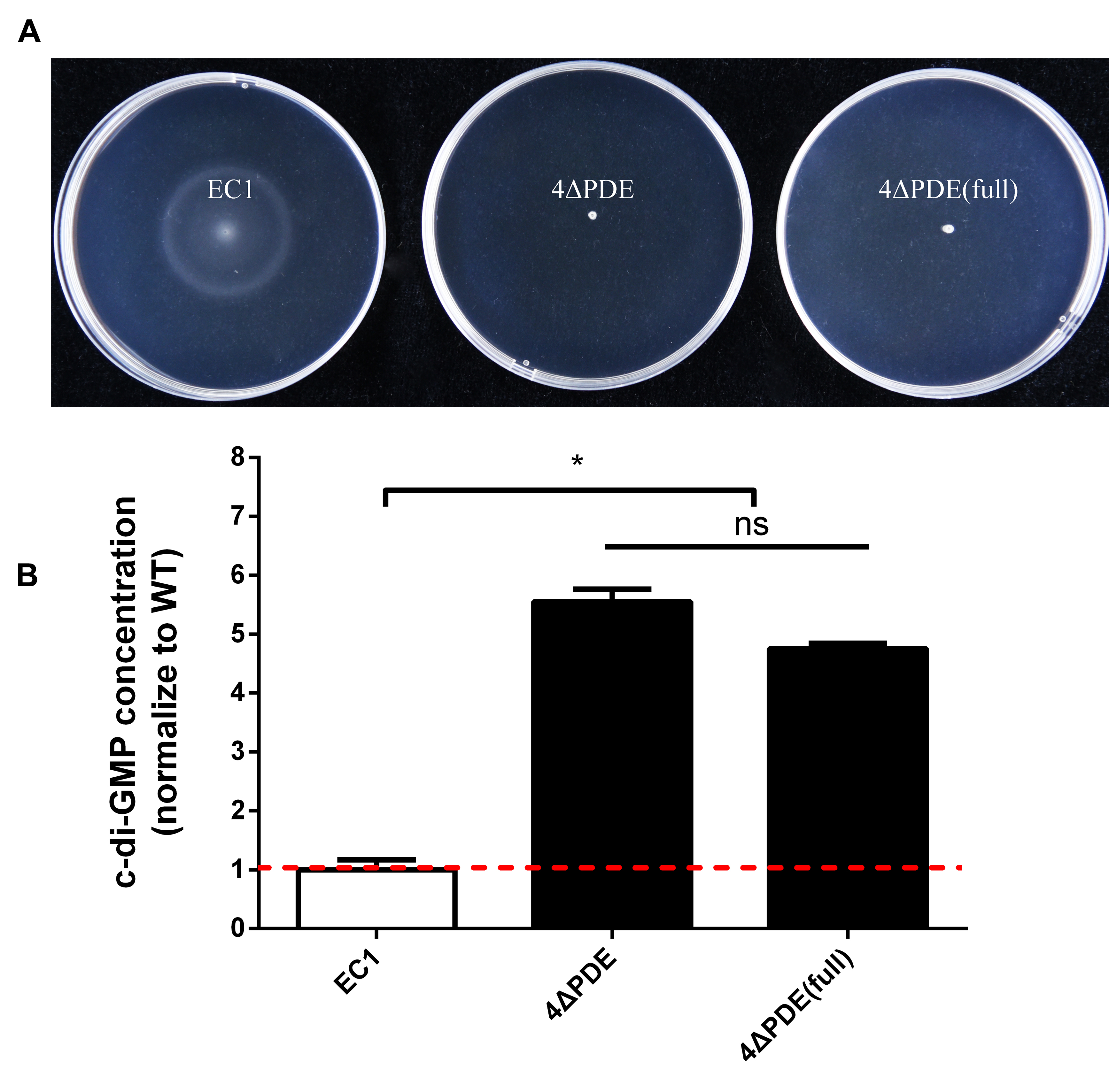

Supplement: FIG S4 [file mBio.02993-20-sf004.jpg]

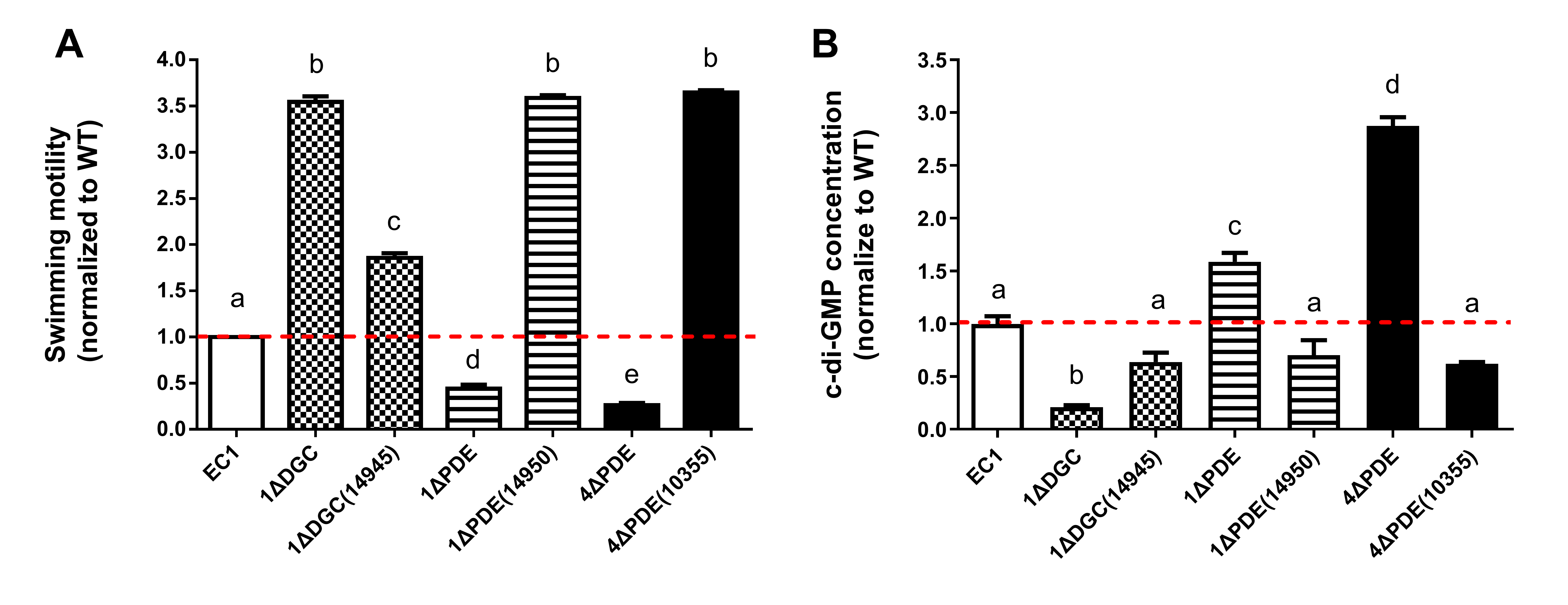

Supplement: FIG S5 [file mBio.02993-20-sf005.tif]

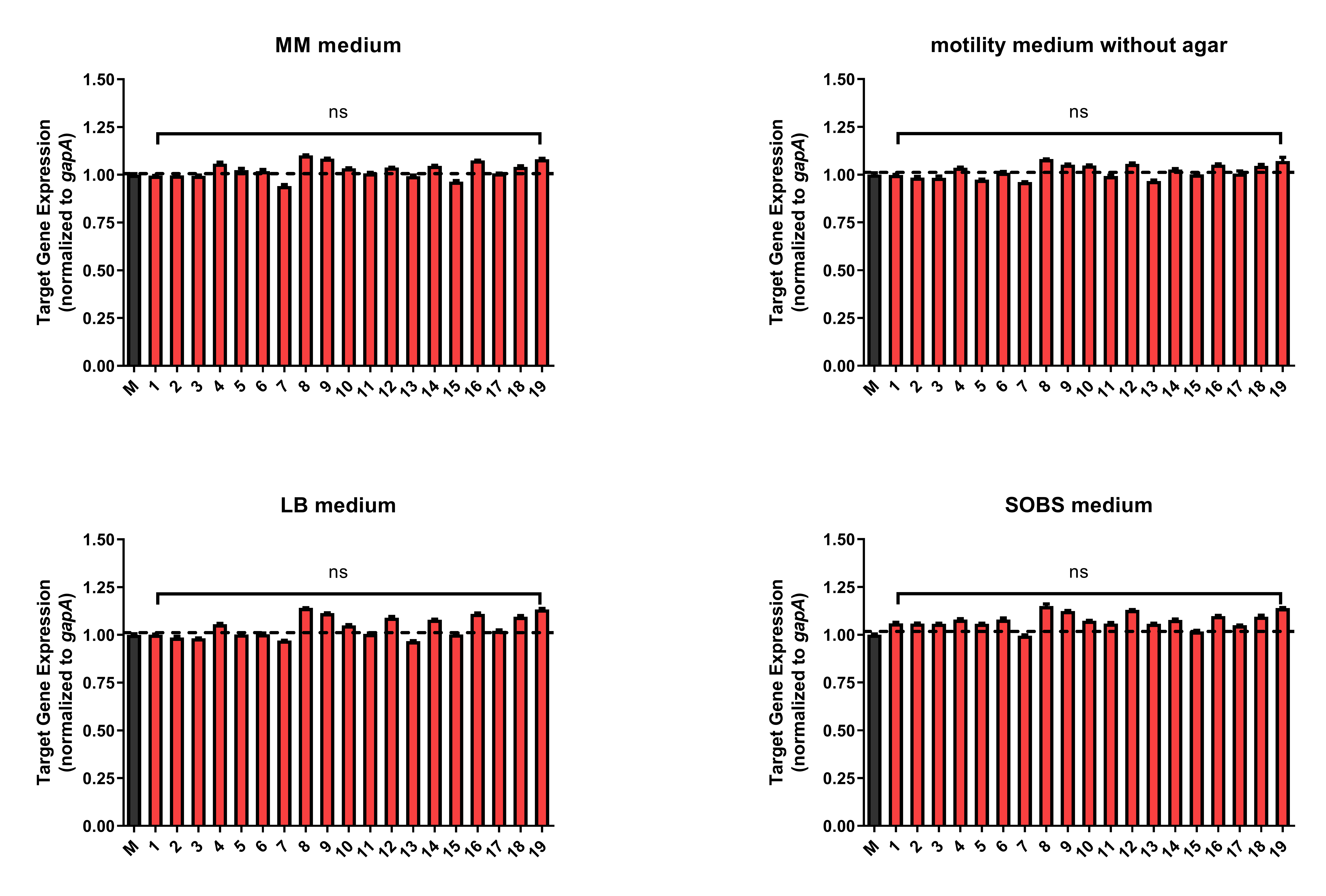

Supplement: FIG S6 [file mBio.02993-20-sf006.tif]

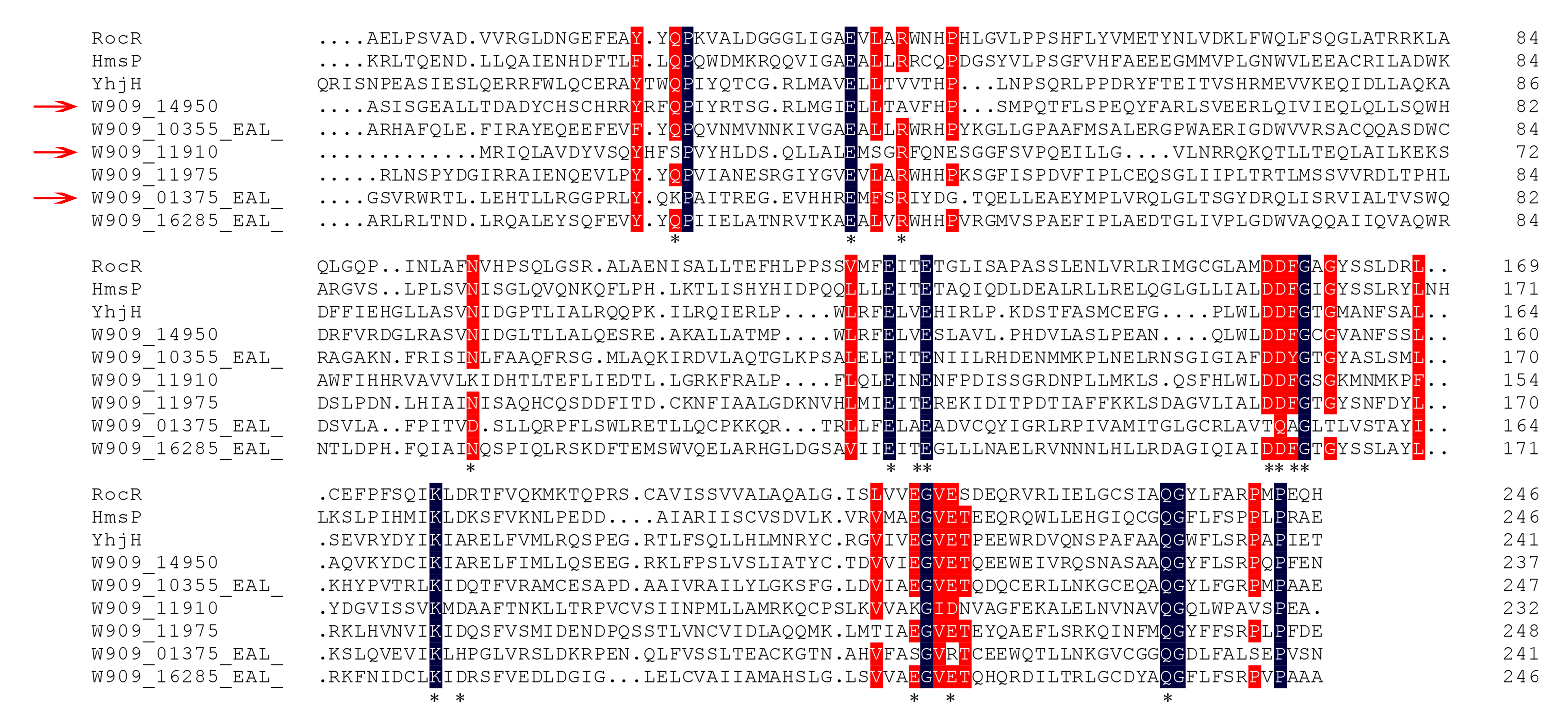

Supplement: FIG S7 [file mBio.02993-20-sf007.tif]

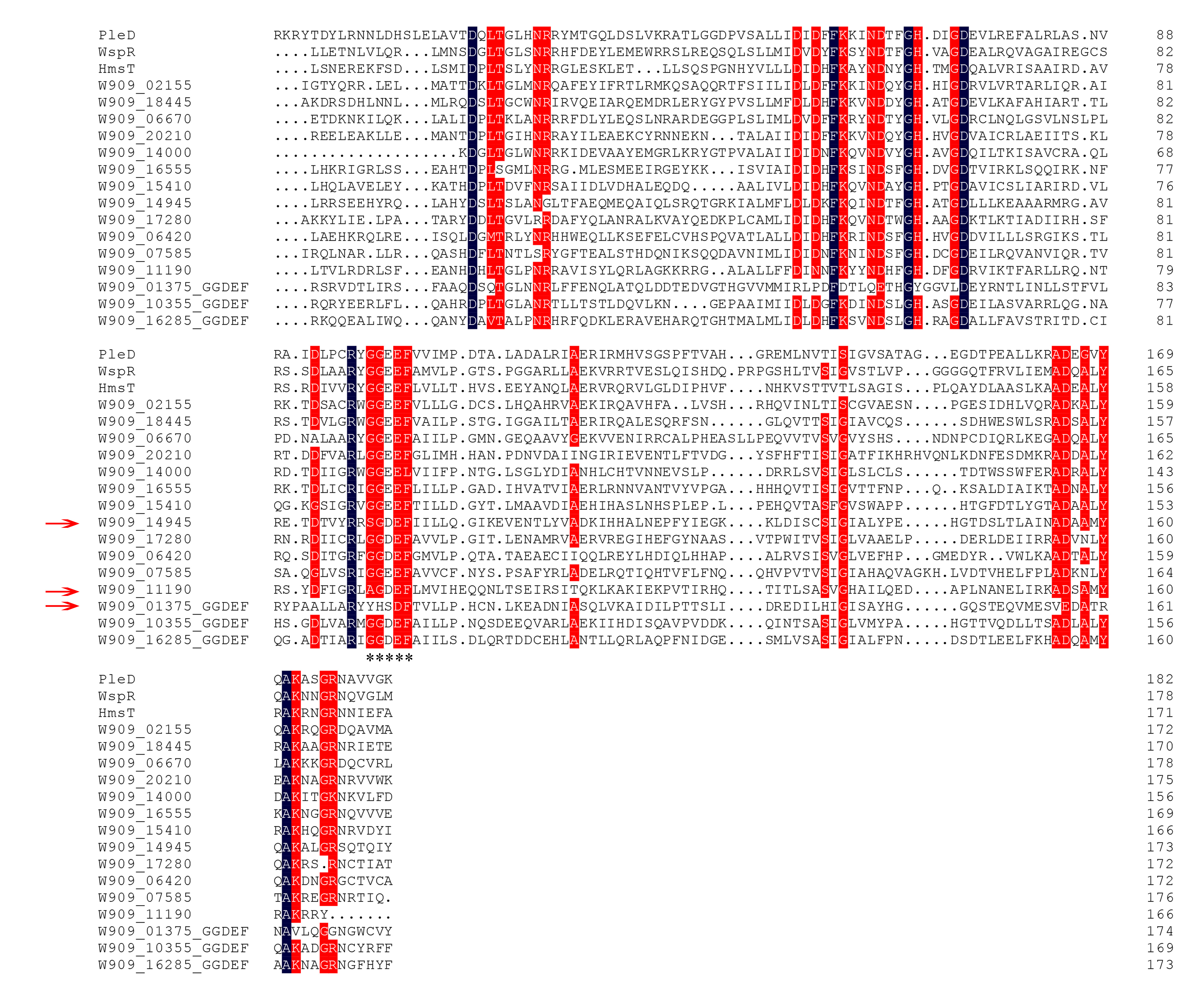

Supplement: FIG S8 [file mBio.02993-20-sf008.jpg]
