# Supplementary material for: Systematic Analysis of c-di-GMP Signaling Mechanisms and Biological Functions in Dickeya zeae EC1
Source: mBio. 2020 Dec 1;11(6):e02993-20. doi: 10.1128/mBio.02993-20 (PMC7733949; doi:10.1128/mBio.02993-20)
Supplement: TABLE S1 [file mBio.02993-20-st001.pdf]

TABLE S1 Strains and plasmids used in this study.

Name of strain	Relevant phenotype	References of source
EC1	Wild-type *D. zeae* EC1, Pm^r	Lab collection
15ΔDGC	Deletion of all c-di-GMP synthesis genes of EC1	This study
7ΔPDE	Deletion of all c-di-GMP degradation genes of EC1	This study
7ΔPDEΔ*W909_08750*	Deletion of all c-di-GMP degradation genes and *W909_08750* of EC1	This study
1ΔDGC	in frame deletion of c-di-GMP synthesis gene*W909_14945* in EC1	This study
2ΔDGC	in frame deletion of c-di-GMP synthesis genes*W909_14945* and *W909_02155* in EC1	This study
3ΔDGC	in frame deletion of c-di-GMP synthesis genes*W909_14945*, *W909_02155* and *W909_0642*0 in EC1	This study
4ΔDGC	in frame deletion of c-di-GMP synthesis genes*W909_14945*, *W909_02155*, *W909_0642*0 and *W909_06670* in EC1	This study
5ΔDGC	in frame deletion of c-di-GMP synthesis genes*W909_14945*, *W909_02155*, *W909_0642*0, *W909_06670* and *W909_07585* in EC1	This study
6ΔDGC	in frame deletion of c-di-GMP synthesis genes*W909_14945*, *W909_02155*, *W909_0642*0, *W909_06670*, *W909_07585* and *W909_11190* in EC1	This study
7ΔDGC	in frame deletion of c-di-GMP synthesis genes*W909_14945*,	This study

	W909_02155, *W909_06420*, *W909_06670*, *W909_07585*, *W909_11190* and *W909_14000* in EC1	
8ΔDGC	in frame deletion of c-di-GMP synthesis genes*W909_14945*, *W909_02155*, *W909_06420*, *W909_06670*, *W909_07585*, *W909_11190*, *W909_14000* and *W909_16555* in EC1	This study
9ΔDGC	in frame deletion of c-di-GMP synthesis genes*W909_14945*, *W909_02155*, *W909_06420*, *W909_06670*, *W909_07585*, *W909_11190*, *W909_14000*, *W909_16555* and *W909_15410* in EC1	This study
10ΔDGC	in frame deletion of c-di-GMP synthesis genes*W909_14945*, *W909_02155*, *W909_06420*, *W909_06670*, *W909_07585*, *W909_11190*, *W909_14000*, *W909_16555*, *W909_15410* and *W909_17280* in EC1	This study
11ΔDGC	in frame deletion of c-di-GMP synthesis genes*W909_14945*, *W909_02155*, *W909_06420*, *W909_06670*, *W909_07585*, *W909_11190*, *W909_14000*, *W909_16555*, *W909_15410*, *W909_17280* and *W909_18445* in EC1	This study
12ΔDGC	in frame deletion of c-di-GMP synthesis genes*W909_14945*, *W909_02155*, *W909_06420*, *W909_06670*, *W909_07585*, *W909_11190*, *W909_14000*, *W909_16555*, *W909_15410*, *W909_17280*, *W909_18445* and *W909_20210* in EC1	This study
13ΔDGC	in frame deletion of c-di-GMP synthesis genes*W909_14945*, *W909_02155*, *W909_06420*, *W909_06670*, *W909_07585*, *W909_11190*, *W909_14000*, *W909_16555*, *W909_15410*, *W909_17280*, *W909_18445*,	This study

	W909_20210 and the GGDEF domain of *W909_01375* in EC1	
14ΔDGC	in frame deletion of c-di-GMP synthesis genes*W909_14945*, *W909_02155*, *W909_0642*0, *W909_06670*, *W909_07585*, *W909_11190*, *W909_14000*, *W909_16555*, *W909_15410*, *W909_17280*, *W909_18445*, *W909_20210*, the GGDEF domain of *W909_01375* and *W909_10355* in EC1	This study
1ΔPDE	in frame deletion of c-di-GMP degradation gene*W909_14950* in EC1	This study
2ΔPDE	in frame deletion of c-di-GMP degradation genes*W909_14950* and *W909_11910* in EC1	This study
3ΔPDE	in frame deletion of c-di-GMP degradation genes*W909_14950*, *W909_11910* and the EAL domain of *W909_16285* in EC1	This study
4ΔPDE	in frame deletion of c-di-GMP degradation genes*W909_14950*, *W909_11910,* the EAL domain of *W909_16285* and *W909_10355* in EC1	This study
5ΔPDE	in frame deletion of c-di-GMP degradation genes*W909_14950*, *W909_11910,* the EAL domain of *W909_16285*, *W909_10355* and *W909_14520* in EC1	This study
6ΔPDE	in frame deletion of c-di-GMP degradation genes*W909_14950*, *W909_11910,* the EAL domain of *W909_16285*, *W909_10355,* *W909_01375* and *W909_14520* in EC1	This study
ΔzmsA	in frame deletion of *zmsA* gene, the most essential gene for production for zeamines, in EC1	This study
ΔfliG	in frame deletion of *fliG* gene, a component of the switch complex on	This study

	the rotor of the bacterial flagellum, in EC1	
ΔbcsA	in frame deletion of *bcsA* gene, one of the key cellulose synthase enzymes involved in biofilm formation, in EC1	This study
EC1 (pBBR1MCS4)	Wild-type *D. zeae* EC1 containing the control plasmid pBBR1-MCS4	This study
15ΔDGC (pBBR1MCS4)	c-di-GMP free strain 15 Δ DGC containing the control plasmid pBBR1-MCS4	This study
7ΔPDE (pBBR1MCS4)	c-di-GMP maximum strain 7 Δ PDE containing the control plasmid pBBR1-MCS4	This study
15ΔDGC (pBBR1-WspR)	15ΔDGC containing a GGDEF domain coding region from *wspR* of PAO1 at the downstream of *lac* promoter, Ap^r	This study
7ΔPDE (pBBR1-RocR)	7ΔPDE containing an EAL domain coding region from *rocR* of PAO1 at the downstream of *lac* promoter, Ap^r	This study
1ΔDGC (14945)	1ΔDGC containing the coding region from *W909_14945* of EC1 at the downstream of *lac* promoter, Ap^r	This study
1ΔPDE (14950)	1ΔPDE containing the coding region from *W909_14950* of EC1 at the downstream of *lac* promoter, Ap^r	This study
4ΔPDE (10355)	4ΔPDE containing the coding region from *W909_10355* of EC1 at the downstream of *lac* promoter, Ap^r	This study
EC1 (GFP)	EC1 containing GFP locus in the broad-host-range cosmid cloning vector pLAFR3, Tc^r	This study
15ΔDGC (GFP)	15 Δ DGC containing GFP locus in the broad-host-range cosmid cloning vector pLAFR3, Tc^r	This study

7ΔPDE (GFP)	7 Δ PDE containing GFP locus in the broad-host-range cosmid cloning vector pLAFR3, Tc^r	This study
ΔzmsA (GFP)	Δ ZmsA containing GFP locus in the broad-host-range cosmid cloning vector pLAFR3, Tc^r	This study
CC118*λ*	*gyrA*，*recA*，*λ pir*	Lab collection
E. coli DH5α	*deoR*, *recA*, *endA*, *hsdR*, *supE*, *thi*, *gyrA*, *relA*	Lab collection
BL21(DE3)	*E. coli* str. *B F– ompT gal dcm lon hsdSB(rB–mB–) λ(DE3 [lacI lacUV5-T7p07 ind1 sam7 nin5]) [malB+]K-12(λS)*	Lab collection

**Name of plasmid**	**Relevant phenotype**	**References of source**
pKNG101	Suicide vector; Str^r, *SacB*, *mobRK2*, *oriR6K* (pir-minus)	Lab collection
pRK2013	Tra^+, Mob^-, ColE1-replicon, Kan^r, Spe^r	Lab collection
pET-28b(+)	Overexpression and purification vector, Kan^r	Lab collection
pET-32a(+)	Overexpression and purification vector, Ap^r	Lab collection
pKNG-01375(EAL)	*W909_01375* knock-out EAL domain fragment ligated on pKNG101	This study
pKNG-01375(GGDEF)	*W909_01375* knock-out GGDEF domain fragment ligated on pKNG101	This study
pKNG-10355(EAL)	*W909_10355* knock-out EAL domain fragment ligated on	This study

	pKNG101	
pKNG-10355(GGDEF)	*W909_10355* knock-out GGDEF domain fragment ligated on pKNG101	This study
pKNG-16285(EAL)	*W909_16285* knock-out EAL domain fragment ligated on pKNG101	This study
pKNG-16285(GGDEF)	*W909_16285* knock-out GGDEF domain fragment ligated on pKNG101	This study
pKNG-08750	*W909_08750* knock-out fragment ligated on pKNG101	This study
pKNG-fliG	*fliG* knock-out fragment ligated on pKNG101	This study
pKNG-bcsA	*bcsA* knock-out fragment ligated on pKNG101	This study
pBBR1-MCS4	Expression vector contains a *lac*Z promoter, Ap^r	Lab collection
pBBR1-WspR	pBBR1-MCS4 carries the coding region of *wspR* at down-stream of *lac* promoter, Ap^r	This study
pBBR1-RocR	pBBR1-MCS4 carries the coding region of *rocR* at down-stream of *lac* promoter, Ap^r	This study
pBBR1-10355	pBBR1-MCS4 carries the coding region of *W909_10355* at down-stream of *lac* promoter, Ap^r	This study
pBBR1-14945	pBBR1-MCS4 carries the coding region of *W909_14945* at down-stream of *lac* promoter, Ap^r	This study
pBBR1-14950	pBBR1-MCS4 carries the coding region of *W909_14950* at down-stream of *lac* promoter, Ap^r	This study

pLAFR3-GFP	broad-host-range cosmid cloning vector pLAFR3 carries the coding region of GFP protein at down-stream of pTAC promoter, Tc^r	This study
