# Supplementary material for: Systematic Analysis of c-di-GMP Signaling Mechanisms and Biological Functions in Dickeya zeae EC1
Source: mBio. 2020 Dec 1;11(6):e02993-20. doi: 10.1128/mBio.02993-20 (PMC7733949; doi:10.1128/mBio.02993-20)
Supplement: TABLE S2 [file mBio.02993-20-st002.pdf]

TABLE S2 Primers used in this study.

Primer name	Primer sequence	Used for plasmid
1-ΔGGDEF-1	cccctgcaggtcgacggatccGATCAGGCTCTGTTGGTTCAGG	pKNG101
1-ΔGGDEF-2	tttccggcacccgtcggtcACGGGCCTCACGCGCTTC	pKNG101
1-ΔGGDEF-3	tGACCGACGGGTGCCGGAA	pKNG101
1-ΔGGDEF-4	cggactatagactatactagtGGCAACCTAGACCCGTAATCAT	pKNG101
1-ΔEAL-1	cccctgcaggtcgacggatccCAAACCGGCTTGAATAATCGG	pKNG101
1-ΔEAL-2	attttttcagcgtACCCCGGCCTTTTTCCGG	pKNG101
1-ΔEAL-3	ccggggtACGCTGAAAAAATATTCACCACG	pKNG101
1-ΔEAL-4	cggactatagactatactagtAGCCACCACGGAAGGCTC	pKNG101
6-ΔGGDEF-1	cccctgcaggtcgacggatccTGTGTTGTGCATCATCGACAACA	pKNG101
6-ΔGGDEF-2	tctcaactcacgggtCCTTTCCGTCACATCACGCA	pKNG101
6-ΔGGDEF-3	aaaggACCCGTGAGTTGAGAGAAGTCTTT	pKNG101
6-ΔGGDEF-4	cggactatagactatactagtTACTGAGTGACGCATAGCCTGTG	pKNG101
6-ΔEAL-1	cccctgcaggtcgacggatccCTTACCGGTCTGGCGAATCG	pKNG101
6-ΔEAL-2	ttctcgaaGGCCTGAAAGACTTCTCTCAACTC	pKNG101
6-ΔEAL-3	gtctttcaggccTTCGAGAAATTGCTCGATCTGG	pKNG101
6-ΔEAL-4	cggactatagactatactagtCATTTTCAAGCATCCATAACCTTTA	pKNG101
16-ΔGGDEF-1	cccctgcaggtcgacggatccCTCGATGAAGGCGGCAAGC	pKNG101
16-ΔGGDEF-2	Tgcgctgggtaatgtcggaa	pKNG101
16-ΔGGDEF-3	ttccgacattacccagcgcACGCCTGCCTTGCAACAG	pKNG101
16-ΔGGDEF-4	cggactatagactatactagtATGCAGATTGTTGTTCACCCG	pKNG101
16-ΔEAL-1	cccctgcaggtcgacggatccGCCAACTACGATGCCGTTACC	pKNG101
16-ΔEAL-2	ttgcagcagcatcgtcaaGGCGTGTGCAGCCTGTTG	pKNG101
16-ΔEAL-3	ccTTGACGATGCTGCTGCAAGC	pKNG101
16-Δ-EAL-4	cggactatagactatactagtGCGGTGGAGCGATTGGTG	pKNG101
07850-1	cccctgcaggtcgacggatccTTTCACACTATCCTGCCGCTT	pKNG101
07850-2	gacttcgcCGTTATTCAGTGTCCCAGCCC	pKNG101
07850-3	cactgaataacgGCGAAGTCAAACAGATAGAGCGC	pKNG101
07850-4	acttatggtacccggggatccAGTCCCATCATTACCACTGGCA	pKNG101
fliG-1	cccctgcaggtcgacggatccCACCTATTTCAACGCCGCC	pKNG101
fliG-2	aCATAGTTCGTTACTCATCCAATGGC	pKNG101
fliG-3	ggatgagtaacgaactatgTAATTCTTCCAGTGATCTTGACTGGC	pKNG101
fliG-4	cggactatagactatactagtTGGAATTCATTGACCATTTGCTG	pKNG101
bcsA-1	cccctgcaggtcgacggatccAAGGCAACAAGCTGACAGGG	pKNG101
bcsA-2	aggtcCAGCACGGCTAAAAACAGGATC	pKNG101
bcsA-3	tttttagccgtgctgGACCTGAAGAAGGAGGACGTTG	pKNG101
bcsA-4	cggactatagactatactagtTGGGAAATTCTCCAGCCGG	pKNG101
PKNG-F	GCCATCAAACCACGTCAAAT	pKNG101
PKNG-R	AACCAAGCCTATGCCTACAG	pKNG101

wspR-Hind III	cccaagcttCATGTACCACTCGCGTTCG	pBBR1-MCS4
wspR-EcoR I	cggaattcTCAGCCCGCCGGGGCC	pBBR1-MCS4
rocR-Hind III	cccaagcttTGTGTGGACCGTGATG	pBBR1-MCS4
rocR-EcoR I	cggaattcTCAGGATCCGGAGCAATAGT	pBBR1-MCS4
10355-EcoR I	gataagcttgatatcgaattcTTTTTTTATCTCTGGCGATGTACG	pBBR1-MCS4
10355-BamH I	cgctctagaactagtggatccATTTGGATAAAACAAGGAAATTGCA	pBBR1-MCS4
14945-EcoR I	gataagcttgatatcgaattcGAGTAAGTCCTCTCGACTCCCGC	pBBR1-MCS4
14945-BamH I	cgctctagaactagtggatccAGCTATCTGCTAAAGCCAATACGA	pBBR1-MCS4
14950-EcoR I	gataagcttgatatcgaattcCAATGCCGTTAGAGAAGGGCT	pBBR1-MCS4
14950-BamH I	cgctctagaactagtggatccTTATACGGTATATTTGGGGGAGATG	pBBR1-MCS4
MCS-F	TCTTCGCTATTACGCCAGCT	pBBR1-MCS4
MCS-R	GGCTCGTATGTTGTGTGGAA	pBBR1-MCS4
14945G(32a)-1	gccatggctgatatcggatccTTGAAAGATATCCTGGATGCGAT	pET-32a
14945(32a)-R	ctcgagtgcggccgcaagcttCTGGTGTGTATTCAGATCATGGTAGA	pET-32a
pET-14950(N+X)-F	taagaaggagatataccatggCAATGGCGGAATACATAGTGCA	pET-28b
pET-14950-R	gtggtggtggtggtgctcgagCAGCTCTGAACGGAGATCTTCC	pET-28b
pET-10355(N+X)-F	taagaaggagatataccatggCCATGAGTCGAGCCCCAGC	pET-28b
pET-10355-R	gtggtggtggtggtgctcgagCCCCAGAGGCGTATCCTCC	pET-28b

**Name of RT-PCR primers**	**Primer sequence**
c-di-GMP(02155)-1	CTCTCCTGAAGATAAGTG
c-di-GMP(02155)-2	AAGTTACCGCTATAATCG
c-di-GMP(06420)-1	GTATTATCCAGCAATTACG
c-di-GMP(06420)-2	CATTATCCTTAGCCTGAT
c-di-GMP(06670)-1	GTATTATCCAGCAATTACG
c-di-GMP(06670)-2	CATTATCCTTAGCCTGAT
c-di-GMP(07585)-1	TGCCAATACCTGAATAAC
c-di-GMP(07585)-2	CGTAGAACATCGTATCTT
c-di-GMP(11190)-1	TCAAATACTATAACGACCAT
c-di-GMP(11190)-2	AATAACCATCAGAAACTCA
c-di-GMP(14000)-1	ATCATCAGATAGAGAATCG
c-di-GMP(14000)-2	CTTCGGTCAGAATATGTT
c-di-GMP(14945)-1	TATGTCGCTGATAAGATTC
c-di-GMP(14945)-2	CGTTAATGGCTAATGTCA
c-di-GMP(15410)-1	CATTTCAAACAGGTCAAC
c-di-GMP(15410)-2	GTAATACATCACGAATGC
c-di-GMP(16555)-1	GATGTGATGCTGAATGTA
c-di-GMP(16555)-2	GCTTGGTGATGTAATAGG
c-di-GMP(17280)-1	ATAATATCATTCGCCTGTA
c-di-GMP(17280)-2	ATAATCGCCATATCATTAAG
c-di-GMP(18445)-1	CGACTCTTCTTGTTACTAT

c-di-GMP(18445)-2	CTTGCCACATTGATAATC
c-di-GMP(20210)-1	CTGCTGGAATTGATTAAC
c-di-GMP(20210)-2	TCTTATTATCGTGCTCTC
c-di-GMP(11910)-1	AATTAAGTCAGTCGTTCC
c-di-GMP(11910)-2	TCATTGCCAGTAACATAG
c-di-GMP(11975)-1	CCACTATCATTTCGCTAT
c-di-GMP(11975)-2	TACCGTCATAAGGAGAAT
c-di-GMP(14950)-1	TTCATTATGCTGCTACAA
c-di-GMP(14950)-2	TACAGTAAGTGGCTATCA
c-di-GMP(14520)-1	TTGAGTGCCAAGATTATG
c-di-GMP(14520)-2	GCGTTACCGATATCATTA
c-di-GMP(01375)-1	CTGATTAACCTGTTGTCT
c-di-GMP(01375)-2	CGATATTATCCGCTTCTT
c-di-GMP(10355)-1	ACTGCTATCGTTTCTTTA
c-di-GMP(10355)-2	TTGTTATTGACCATATTGAC
c-di-GMP(16285)-1	ACAGATTTCAGGATAAACT
c-di-GMP(16285)-2	AGGTCTATCAGCATTAAC

**Name of qPCR primers**	**Primer sequence**
fliG(qPCR)-1	TCTTGTTAATGACCATCG
fliG(qPCR)-2	CAATACTTCCAGCAACTC
fliM(qPCR)-1	GCCGAGAATTTACACATA
fliM(qPCR)-2	GTGGTGATATTGGTGAAT
fliN(qPCR)-1	AAATGACGATCAAAGAGC
fliN(qPCR)-2	GTTCTGATGGTGTAATAATATC
rpoS(qPCR)-1	CTGGACTTGATTGAAGAG
rpoS(qPCR)-2	TTGACGATATGGATAGGT
dksA(qPCR)-1	AAGTCGATAGAACCGTAT
dksA(qPCR)-2	CTCACAATAGCCAAAGTC
bcsA(qPCR)-1	TCTGGATTGTGTTGTTAT
bcsA(qPCR)-2	TCGTTATAGGTAGGAATATAC
bcsB(qPCR)-1	AGGAGAACTATACGCTTA
bcsB(qPCR)-2	AATCTGACTCTTGATATTGT
bcsC(qPCR)-1	TGTAGGTTATCAGTCTTATTCG
bcsC(qPCR)-2	TAGTCACAGCATCATCCA
bcsD(qPCR)-1	GAGATGAATCAGGTATTGT
bcsD(qPCR)-2	GGCAATGACAGATGATAA
YcgR(qPCR)-1	TAGTGGATGACAATATGA
YcgR(qPCR)-2	AGGATCTTACTGATGAAT
flhC(qPCR)-1	GAACATCCACTCTTCCAT
flhC(qPCR)-2	GCGATAAGCCTTGATAAC
flhD(qPCR)-1	GGGTATCAATGAGGAAAT

flhD(qPCR)-2	GGATTCTTGTGTTAATAGC
fliA(qPCR)-1	ACATCTCGTTAGAGGAATATCG
fliA(qPCR)-2	CGCCACTCATCGTAAGAA
flgM(qPCR)-1	AAAGTTAAAGCGTAAAGAGGAA
flgM(qPCR)-2	TTAGACTGAGCGTCACTC
rpoD(qPCR)-1	CCTGCGTCTGGTTATCTC
rpoD(qPCR)-2	ATGTTGCCTTCCTGAATCA
